# A metabolic-to-inflammatory pattern in cardiovascular-kidney-metabolic syndrome staging: a comparative cross-sectional study

**DOI:** 10.3389/fendo.2026.1805355

**Published:** 2026-04-22

**Authors:** Teng Zhang, Zi-Yue Man, Shi-Meng Zhang, Jian-Jun Mu, Yue-Yuan Liao

**Affiliations:** 1 Department of Cardiovascular Medicine, First Affiliated Hospital of Xi’an Jiaotong University, Xi’an, China; 2 Key Laboratory of Molecular Cardiology of Shaanxi Province, Xi’an, China; 3 International Joint Research Center for Cardiovascular Precision Medicine of Shaanxi Province, Xi’an, China; 4 Department of Cardiovascular Medicine, Shaanxi Provincial Second People’s Hospital, Xi’an, China; 5 Department of lmaging, First Affiliated Hospital of Xi’an Jiaotong University, Xi’an, China

**Keywords:** cardiovascular-kidney-metabolic syndrome, cross-sectional study, inflammation, insulin resistance, visceral adiposity

## Abstract

**Background:**

Amid the cardiovascular–kidney–metabolic (CKM) syndrome public health crisis, this study aimed to explore differential pathological associations with established versus advanced CKM status and assess its consistency across Chinese and U.S. adults.

**Methods:**

This cross-sectional study analyzed data from two independent sources: a community survey in Shaanxi, China (n=2, 100) and the U.S. National Health and Nutrition Examination Survey (NHANES) (2011–2018; n=5, 359). Associations between three pathological axes—visceral adiposity (VA), insulin resistance/dyslipidemia pathological score (IRD-PS), and systemic low-grade inflammation pathological score (SLI-PS)—and two key CKM outcomes (established: Stages 2–4 vs. 0–1; advanced: Stages 3–4 vs. 0–2) were evaluated using Firth’s penalized logistic regression to address potential quasi-complete separation in the outcome data.

**Results:**

IRD-PS exhibited the strongest association with established CKM status in both populations (Shaanxi: *OR* = 2.49, 95% *CI* 2.18, 2.86; NHANES: *OR* = 2.52, 95% *CI* 2.24, 2.82). In contrast, SLI-PS was significantly and consistently correlated with advanced CKM status (Shaanxi: *OR* = 1.11, 95% *CI* 1.03, 1.19; NHANES: *OR* = 1.07, 95% *CI* 1.01, 1.14). Statistical exploratory decomposition analysis revealed IRD-PS largely attenuated the association between VA and established CKM status, statistically accounting for 67.1% of the effect in Shaanxi and 64.5% in NHANES. In the NHANES, significant racial/ethnic heterogeneity was observed in the IRD-PS–established CKM status association (*P* for interaction < 0.001), with the strongest association in non-Hispanic Asian people (*OR* = 3.96) and the weakest in non-Hispanic Black people (*OR* = 2.03). These cross-sectional associations should be interpreted with caution.

**Conclusions:**

Our findings support a conceptual model of CKM syndrome in which metabolic dysregulation is the primary correlate of the established (Stage 2+) CKM status and in which systemic inflammation is a more prominent correlate of advanced (Stage 3+) CKM status. These findings may primarily generalize to the analyzed subsamples rather than broader general populations. Given the cross-sectional design, all findings are strictly hypothesis-generating, and the proposed stage-specific pathological pattern requires formal validation in future longitudinal cohorts.

## Introduction

Cardiovascular–kidney–metabolic (CKM) syndrome, defined by the intricate interconnections among obesity, metabolic dysregulation, chronic kidney disease (CKD), and cardiovascular disease (CVD), represents an emerging public health crisis (1). Noncommunicable diseases (NCDs) are responsible for approximately 75% of global deaths, with CKM syndrome playing a substantial role (2). The contributing factors include lifestyle elements such as physical inactivity and suboptimal nutrition, in addition to genetic susceptibilities, which collectively promote long-term health decline (3). Established by the American Heart Association in 2023, CKM syndrome constitutes a progressive spectrum that necessitates timely interventions (1). Common underlying mechanisms, including chronic inflammation, insulin resistance, and endothelial dysfunction, unify its constituent disorders (4). With more than 2.1 billion adults globally affected by overweight or obesity, the burden of CKD and CVD is projected to nearly double by 2050, driven by urbanization and demographic aging (5).

Heterogeneity in metabolic phenotypes challenges the conventional link between obesity and CVD. Metabolically Healthy Obese (MHO) individuals may transiently exhibit relatively preserved insulin sensitivity amid excess fat mass, whereas Metabolically Unhealthy Non-Obese (MUNO) individuals display metabolic disturbances in the absence of overt obesity (6). Standard metabolic syndrome (MetS) diagnostic criteria inadequately address these subtleties, limiting their effectiveness in pinpointing phenotype- and stage-specific contributors (7). Visceral adiposity (VA) serves as a pivotal biological link, as it results in the secretion of adipokines that promote insulin resistance and dyslipidemia, thereby aggravating CKD and CVD progression (8). Likewise, insulin resistance, dyslipidemia, and proinflammatory states establish reinforcing cycles that intensify endothelial damage (9, 10).

Despite its wide clinical use, the traditional MetS framework has critical limitations for guiding staged, precision management. MetS is defined as a binary categorical construct based on the co-occurrence of 3 or more risk factors. This fails to capture the progressive, continuum nature of cardiometabolic and renal damage, and cannot distinguish between early risk factor accumulation, established metabolic disease, and advanced organ impairment (1, 11). In contrast, the 2023 AHA CKM syndrome staging system provides a standardized, hierarchical framework that stratifies patients from at-risk states (Stages 0–1) to established disease (Stage 2) and advanced organ damage (Stages 3–4). This staging system not only unifies the interconnected pathological processes across cardiovascular, renal, and metabolic systems, but also enables stage-specific risk stratification and targeted intervention (12).

Existing research offers fragmented perspectives on CKM pathophysiology. For example, the Framingham Heart Study demonstrated that visceral fat accumulation is strongly associated with multiple cardiometabolic risk factors, independent of overall adiposity (13), and mediation analyses in cohorts with diabetes have revealed that inflammation contributes to renal function deterioration (14). Nevertheless, numerous investigations depend on univariate approaches and overlook the multidimensional dynamics of CKM pathways. To overcome these limitations, our study constructed three standardized pathological axes: visceral adiposity (VA), an insulin resistance/dyslipidemia pathological score (IRD-PS), and a systemic low-grade inflammation pathological score (SLI-PS). The latter two axes were generated by consolidating correlated biomarkers using principal component analysis (PCA), a method previously used in MetS groupings (15). Using this multidimensional framework, we hypothesized that distinct pathological processes are differentially associated with established versus advanced CKM status across diverse metabolic phenotypes.

In this cross-sectional study, the associations of VA, IRD-PS, and SLI-PS with established and advanced CKM status were investigated in a community-based sample from Shaanxi, China, and a multiethnic U.S. sample from the National Health and Nutrition Examination Survey (NHANES). A key secondary objective was to determine whether this pattern of stage-specific associations remains stable when tested in different populations using distinct, commonly available clinical measurement tools. By comparing these patterns, we seek to provide insights for future precision prevention strategies that are more equitable and effective across an increasingly diverse, globalized, and aging populace.

Specifically, we tested the following *a priori* hypotheses:

1. Metabolic dysregulation would relate primarily to established CKM status (Stages 2–4 vs. 0–1), whereas systemic inflammation would be more strongly associated with advanced CKM status (Stages 3–4 vs. 0–2), with such patterns expected to be consistent between Chinese and U.S. populations.
2. The association between visceral adiposity and CKM status would be stage-specific: attenuated by metabolic profiles among individuals with established CKM status, but attenuated by inflammatory profiles in those with advanced CKM status.
3. The magnitude and pattern of these stage-specific associations, as well as the underlying patterns of statistical attenuation, would differ significantly across age, sex, and race/ethnicity subgroups.

## Methods

### Study design and population

This cross-sectional study utilized data from two independent sources: a primary discovery sample from a 2022 community-based survey in Shaanxi, China, and an external exploratory sample from the U.S. National Health and Nutrition Examination Survey (NHANES) 2011–2018, a nationally representative health survey.

From an initial 9, 336 nonpregnant adults (≥ 18 years) in Shaanxi, several communities were randomly selected to form a nested sub-sample, and 2, 179 residents in these selected communities with complete echocardiography and brachial-ankle pulse wave velocity (baPWV) data were selected. After the exclusion of 79 participants with missing key data, acute infections, or recent cardiovascular events, the final analytical sample comprised 2, 100 individuals. A comparison of baseline characteristics between these included participants and the 7, 236 excluded individuals revealed significant differences in age, anthropometrics, and metabolic profiles (**Supplementary Table 1**).

The exploratory sample was derived from four continuous cycles of the NHANES 2011–2018, encompassing 39, 156 enrolled participants (2011–2012: n = 9, 756; 2013–2014: n = 10, 175; 2015–2016: n = 9, 971; 2017–2018: n = 9, 254). After excluding participants with missing data on visceral fat or CKM staging components (**Supplementary Figure 1**), the final analytical sample comprised 5, 359 non-pregnant adults aged ≥20 years, distributed across cycles as follows: 1, 282 (2011–2012), 1, 532 (2013–2014), 1, 385 (2015–2016), and 1, 160 (2017–2018). Similarly, this included sub-sample differed significantly from the 17, 258 excluded adults (≥ 20 years), particularly in age and lifestyle factors (**Supplementary Table 2**).

This study was approved by the respective ethics committees (Ethics Committee of Fuwai Hospital, Approval No. 2020-1360, and National Center for Health Statistics Research Ethics Review Board). All participants provided written informed consent, and both studies adhered to the principles of the Declaration of Helsinki.

### Data collection and clinical measurements

For the Shaanxi dataset, data on sociodemographics, lifestyle patterns, and health markers were collected via interviewer-administered questionnaires. All measurements were performed in the morning after an overnight fast by trained and certified technicians following a standardized protocol. Participants were instructed to avoid strenuous exercise, smoking, and alcoholic or caffeinated beverages for at least 30 minutes prior to the examinations. A strict measurement sequence was followed, with blood pressure and ABI measurements conducted before phlebotomy or at least 30 minutes after. The detailed protocols for the anthropometric, hemodynamic, and cardiovascular assessments are described in **Supplementary Methods S1**-**S3**.

The National Center for Health Statistics (NCHS) collected NHANES data via standardized household interviews and performed physical examinations and laboratory tests at mobile examination centers. Specific details about the methodology are available on the website (https://www.cdc.gov/nchs/nhanes/index.html).

### Definitions of key variables in Shaanxi sample

#### 1. Metabolic phenotypes and metabolic syndrome

Participants were categorized into four phenotypes by combining their weight status (nonobese: BMI < 24 kg/m^2^ vs. obese: BMI ≥ 24 kg/m^2^, using Chinese criteria (16)) and their metabolic health (healthy: < 3 MetS components vs. unhealthy: ≥ 3 MetS components). This resulted in four groups: Metabolically Healthy Non-Obese (MHNO), Metabolically Unhealthy Non-Obese (MUNO), Metabolically Healthy Obese (MHO), and Metabolically Unhealthy Obese (MUO).

We used a modified 5-component definition of MetS established by the Chinese Diabetes Society (CDS) in 2020: 1) elevated blood pressure (systolic ≥ 130 mmHg, diastolic ≥ 85 mmHg, or antihypertensive medication use); 2) hyperglycemia (fasting glucose ≥ 6.1 mmol/L or antidiabetic medication use); 3) hypertriglyceridemia (TG ≥ 1.7 mmol/L); 4) reduced high-density lipoprotein cholesterol (HDL-C) < 1.04 mmol/L; and 5) abdominal obesity (waist circumference ≥ 90 cm in men or ≥ 85 cm in women).

#### 2. CKM stages

Staging was adapted from the 2023 American Heart Association guidelines (1), with modifications to accommodate the specific variables available in our dataset. For all the analyses, the stages were grouped into three categories: Early (Stages 0–1), Mid (Stage 2), and Late (Stages 3–4).

- Stage 0: No CKM risk factors.
- Stage 1: Presence of excess body fat (BMI ≥ 24 kg/m²) or prediabetes (5.6–6.9 mmol/L fasting glucose level or 5.7–6.4% HbA1c level).
- Stage 2: Presence of hypertension, type 2 diabetes, dyslipidemia, or moderate-to-high-risk chronic kidney disease (CKD) (details in **Supplementary Methods S4**).
- Stage 3: Subclinical cardiovascular disease (CVD), defined as arterial stiffness (brachial-ankle pulse wave velocity [baPWV] ≥ 1800 cm/s), cardiac dysfunction, very high CKD risk, or a high predicted 10-year CVD risk using the AHA’s PREVENT equations.
- Stage 4: Established clinical CVD (self-reported history of coronary events, stroke, or heart failure).

#### 3. Organ-specific outcomes

- Arterial Stiffness: baPWV ≥ 1800 cm/s (17).
- Cardiac Dysfunction: Either systolic dysfunction (left ventricular ejection fraction [LVEF] < 50%) or diastolic dysfunction (E/A ratio ≤ 0.8 or ≥ 2.0 with LVEF ≥ 50%) (18).
- Albuminuria: A urinary albumin-to-creatinine ratio (UACR) ≥ 30 mg/g (19).

### Derivation of pathological axes in Shaanxi sample

To quantify key pathological processes, three standardized axes were derived. Two of these axes were constructed using principal component analysis (PCA), while the visceral adiposity (VA) axis was based on a single standardized measure.

Specifically, the insulin resistance/dyslipidemia pathological score (IRD-PS) and the systemic low-grade inflammation pathological score (SLI-PS) were derived from the first principal component of a PCA with varimax rotation on z-score standardized variables. The IRD-PS combined the triglyceride–glucose (TyG) index, triglyceride (TG)/high density lipoprotein cholesterol (HDL-C) ratio, and HDL-C (negated for directional consistency) values. The SLI-PS combined white blood cell (WBC) count, neutrophil (NE) count, platelet (PLT) count, and uric acid (UA) concentration. Detailed PCA results, including eigenvalues, variance explained, and factor loadings for both scores in the Shaanxi and NHANES datasets, are presented in **Supplementary Table 3**, with specific formulas for the derived variables provided in **Supplementary Methods S4**.

The VA axis was based on the visceral fat grade, which is measured by bioelectrical impedance (InBody 770, InBody Co., Ltd., Seoul, Korea) on an ordinal scale from 1 to 20 (20). To ensure its comparability with the other axes, this grade was also z-score standardized for all analyses.

### NHANES: variable definitions and adaptations

To test the robustness and generalizability of our findings from the primary sample, all key definitions and analytical constructs were systematically re-evaluated in the NHANES 2011–2018 dataset. We aimed for conceptual consistency while intentionally leveraging the different measurement protocols in the NHANES to assess whether our proposed two-stage framework holds true under varied clinical assessment scenarios.

The four metabolic phenotypes (MHNO, MUNO, MHO, and MUO) were reconstructed using U.S.-specific BMI criteria (< 25 kg/m² for nonobese) and the definition of metabolic syndrome (Mets) as per the National Cholesterol Education Program Adult Treatment Panel III (ATP III) criteria (21). CKM staging followed the same principles as in the Shaanxi population, adapted from the 2023 AHA guidelines using available NHANES data (22) (details in **Supplementary Methods S4****).** The three pathological axes were also reconstructed to represent the same underlying biological constructs:

- Visceral adiposity (VA) Axis: Based on the log-transformed visceral fat area measured by dual-energy X-ray absorptiometry (DXA) to account for its skewed distribution. Note that the BIA-derived visceral fat grade used in the Shaanxi sample and the DXA-derived visceral fat area used in the NHANES are conceptually aligned to quantify visceral adiposity, but they are not directly equivalent in measurement metrics, which precludes direct comparison of the absolute parameter estimates between the two samples.
- Insulin resistance/dyslipidemia pathological score (IRD-PS): Combined the homeostatic model assessment for insulin resistance (HOMA-IR), the triglyceride–glucose (TyG) index, and the TG/HDL-C ratio.
- Systemic low-grade inflammation pathological score (SLI-PS): Combined white blood cell (WBC) count, the neutrophil-to-lymphocyte ratio (NLR), and the platelet-to-lymphocyte ratio (PLR).

The variables used to operationalize each construct in the NHANES are further detailed in **Supplementary Methods S4**.

### Statistical analysis

Continuous variables were presented as medians (interquartile ranges), and categorical variables were presented as frequencies (percentages). Differences across groups were assessed using the Kruskal–Wallis test or chi–square test. Additionally, standardized mean differences (SMDs) were calculated to evaluate baseline differences between the included and excluded participants due to missing data, with an SMD < 0.1 considered indicative of a well-balanced covariate. All primary association and exploratory decomposition analyses were based on complete-case analysis (CCA) for both datasets, with only participants having complete core clinical, demographic and outcome data included in the final analytical samples. For the NHANES, all analyses strictly incorporated the complex survey design. To account for the multi-year cycle (2011–2018), a new set of analytical weights was constructed by dividing the original 2-year Mobile Examination Center (MEC) weights by 4, as per National Center for Health Statistics (NCHS) recommendations.

The primary analysis modeled two key binary outcomes: established CKM status (defined as Mid/Late vs. Early stages) and advanced CKM status (Late vs. Early/Mid stages). Firth’s penalized logistic regression was employed for all association analyses to address violation of the proportional odds assumption and quasi-complete data separation. For the Shaanxi sample, inverse probability weighting (IPW) was further implemented as a sensitivity analysis to mitigate potential selection bias and improve the generalizability of findings from the nested sub-sample to the entire survey population (n=9, 336). Stabilized IPW weights were calculated based on the demographic and lifestyle characteristics of the overall Shaanxi population.

A series of nested models were developed for both populations. Model 1 was unadjusted. Model 2 was adjusted for age and sex. The fully adjusted Model 3 further included smoking status, drinking status, physical activity and education level, with a family history of cardiovascular disease or stroke specific to the Shaanxi sample and race/ethnicity specific to the NHANES sample.

To explore dose–response relationships, each pathological axis was categorized into quartiles. We explicitly acknowledge that potential circularity in the primary analysis—arising from the definitional overlap between the lipid components of the IRD-PS and the dyslipidemia criterion for CKM Stage 2—remains an inherent limitation. To address this robustness concern, we performed a specific sensitivity analysis redefining the Established CKM status outcome by excluding participants who were classified as Stage 2 solely due to dyslipidemia. The joint effects of these axes, categorized by their medians, were also assessed. Furthermore, exploratory decomposition analysis with 1, 000 bootstrap simulations was performed to quantify the proportion of the VA-CKM association statistically attenuated by the IRD-PS and SLI-PS. However, the cross-sectional design of this study cannot confirm the temporal sequence of the pathological processes, and residual unmeasured confounding may exist; thus, these exploratory findings only reflect statistical attenuation of associations, rather than definitive causal mediation. Subgroup analyses were stratified by age and sex in the Shaanxi sample and additionally by race/ethnicity in the NHANES. *P* values for interactions were determined using likelihood ratio tests between nested Firth models. All *P* values for interactions were subsequently adjusted using the Benjamini–Hochberg false discovery rate (FDR) method.

All the statistical analyses were performed using R (version 4.5.1), and a two-sided *P* value < 0.05 was considered to indicate statistical significance.

## Results

### Study populations and construction of pathological axes

The analysis included 2, 100 participants from a community-based sample in Shaanxi, China (median age 42.0 years; 45.5% male) and 5, 359 from the U.S. National Health and Nutrition Examination Survey (NHANES) 2011–2018 (median age 41.0 years; 52.0% male). In the Shaanxi sample, stratification by metabolic phenotype revealed significant differences (*P* < 0.001) (**Table 1**). For example, compared with the Metabolically Healthy Non-Obese (MHNO) group, the Metabolically Unhealthy Obese (MUO) group had markedly higher median systolic blood pressure (143 vs. 116 mmHg) and triglyceride levels (2.54 vs. 0.98 mmol/L). The NHANES sample, a multiethnic U.S. population, was composed of approximately 63% non-Hispanic White people, 11% non-Hispanic Black people, and 9.7% Mexican American participants (**Supplementary Table 4**).

**Table 1:** Study population characteristics stratified by metabolically healthy obese phenotype in Shaanxi dataset.

Characteristic	Overall (n = 2, 100)	MHNO (n = 1, 149)	MUNO (n = 179)	MHO (n = 522)	MUO (n = 250)	*P* value	SMD
Sociodemographic Characteristics							
Age (years)	42 (31, 55)	40 (28, 52)	55 (44, 65)	40 (30, 50)	51 (42, 61)	**<0.001**	0.533
Sex (Male), n (%)	955 (45.5%)	446 (38.8%)	95 (53.1%)	255 (48.9%)	159 (63.6%)	**<0.001**	0.267
Education Level, n (%)						**<0.001**	0.410
Primary or below	770 (36.7%)	383 (33.3%)	97 (54.2%)	161 (30.8%)	129 (51.6%)		
Junior high graduation	471 (22.4%)	239 (20.8%)	44 (24.6%)	129 (24.7%)	59 (23.6%)		
Secondary graduate	303 (14.4%)	175 (15.2%)	15 (8.4%)	79 (15.1%)	34 (13.6%)		
Junior college and above	556 (26.5%)	352 (30.6%)	23 (12.8%)	153 (29.3%)	28 (11.2%)		
Lifestyle Factors							
Lack of activity, n(%)	346 (16.5%)	187 (16.3%)	30 (16.8%)	105 (20.2%)	24 (9.6%)	**0.003**	0.153
Current Smoker, n (%)	296 (14.1%)	149 (13.0%)	35 (19.6%)	59 (11.3%)	53 (21.2%)	**<0.001**	0.165
Drinker, n (%)	392 (18.7%)	183 (15.9%)	39 (21.8%)	103 (19.8%)	67 (26.8%)	**<0.001**	0.142
Anthropometrics							
Height (cm)	162.00 (157.50, 169.00)	162.00 (158.00, 168.00)	162.00 (157.00, 170.00)	162.00 (157.00, 169.00)	164.25 (159.00, 170.00)	0.055	0.063
BMI (kg/m²)	22.87 (20.63, 25.30)	21.23 (19.60, 22.62)	22.02 (20.47, 23.09)	25.93 (24.87, 27.64)	26.86 (25.31, 28.57)	**<0.001**	1.939
Waist (cm)	80.00 (75.00, 88.00)	78.00 (72.20, 83.00)	82.10 (78.05, 88.70)	86.00 (80.00, 91.00)	89.40 (81.30, 96.00)	**<0.001**	0.739
Laboratory Tests							
TC (mmol/L)	4.27 (3.75, 4.86)	4.15 (3.67, 4.65)	4.59 (3.87, 5.30)	4.33 (3.81, 4.89)	4.70 (4.08, 5.38)	**<0.001**	0.359
TG (mmol/L)	1.25 (0.85, 1.97)	0.98 (0.71, 1.40)	2.38 (1.79, 3.44)	1.31 (0.96, 1.81)	2.54 (1.97, 3.82)	**<0.001**	0.941
HDL-C (mmol/L)	1.37 (1.21, 1.57)	1.45 (1.29, 1.64)	1.24 (1.03, 1.46)	1.34 (1.20, 1.49)	1.22 (1.04, 1.37)	**<0.001**	0.527
LDL-C (mmol/L)	2.42 (2.01, 2.92)	2.31 (1.92, 2.75)	2.48 (1.94, 3.01)	2.56 (2.16, 3.02)	2.74 (2.18, 3.20)	**<0.001**	0.272
Glucose (mmol/L)	4.94 (4.60, 5.44)	4.82 (4.54, 5.19)	5.48 (4.80, 7.45)	4.92 (4.62, 5.34)	5.60 (4.95, 7.35)	**<0.001**	0.588
HbA1c (%)	5.40 (5.20, 5.70)	5.40 (5.10, 5.60)	5.70 (5.30, 6.10)	5.45 (5.10, 5.70)	5.70 (5.32, 6.30)	**<0.001**	0.479
Cr (μmol/L)	61.00 (53.00, 73.00)	59.00 (52.00, 70.00)	62.00 (53.50, 74.00)	63.00 (53.00, 74.00)	69.00 (57.00, 81.75)	**<0.001**	0.278
UA (μmol/L)	320.00 (266.00, 384.25)	299.00 (252.00, 356.00)	334.00 (275.50, 398.00)	340.00 (279.00, 401.00)	375.00 (312.25, 445.50)	**<0.001**	0.422
UACR (mg/g)	3.91 (1.88, 8.51)	3.56 (1.72, 6.32)	5.80 (2.46, 20.56)	3.90 (1.88, 8.06)	7.74 (3.11, 21.22)	**<0.001**	0.259
WBC (×10^9^/L)	5.88 (4.95, 6.80)	5.69 (4.79, 6.62)	6.01 (5.28, 6.93)	6.00 (5.07, 6.86)	6.36 (5.44, 7.34)	**<0.001**	0.231
TyG index	8.52 (8.09, 9.07)	8.27 (7.91, 8.63)	9.29 (8.95, 9.74)	8.57 (8.20, 8.94)	9.41 (9.08, 9.85)	**<0.001**	1.258
Instrument-based Examination							
SBP (mmHg)	122 (111, 136)	116 (107, 127)	141 (127, 153)	122 (113, 132)	143 (131, 157)	**<0.001**	0.830
DBP (mmHg)	79 (72, 87)	76 (70, 82)	85 (77, 94)	80 (73, 87)	91 (83, 99)	**<0.001**	0.755
VA Grade	8 (6, 11)	6 (5, 8)	7 (6, 8)	11 (9, 13)	11 (9, 14)	**<0.001**	1.207
EF (%)	67.29 (62.64, 71.43)	67.42 (62.79, 71.68)	65.69 (60.77, 69.93)	67.69 (63.15, 71.53)	67.01 (62.57, 71.65)	**0.005**	0.148
baPWV (cm/s)	1256.25 (1146.00, 1448.25)	1204.00 (1114.00, 1349.00)	1489.00 (1260.75, 1708.00)	1247.00 (1158.38, 1427.12)	1507.50 (1362.62, 1713.50)	**<0.001**	0.667
ABI	1.07 (1.02, 1.12)	1.08 (1.03, 1.14)	1.05 (1.00, 1.10)	1.06 (1.01, 1.12)	1.04 (0.99, 1.10)	**<0.001**	0.239
eGFR (mL/min/1.73m²)	124.61 (110.03, 139.60)	129.29 (113.22, 143.65)	112.92 (106.08, 126.22)	126.66 (113.49, 138.75)	112.49 (100.58, 122.75)	**<0.001**	0.565
LVMI (g/m²)	78.56 (68.01, 92.41)	75.98 (66.21, 88.19)	86.95 (76.76, 105.51)	78.65 (69.50, 91.58)	86.41 (73.82, 104.95)	**<0.001**	0.225
Disease History/Medication							
Hypertension, n (%)	608 (29.0%)	154 (13.4%)	124 (69.3%)	121 (23.2%)	209 (83.6%)	**<0.001**	1.085
Diabetes, n (%)	204 (9.7%)	30 (2.6%)	67 (37.4%)	13 (2.5%)	94 (37.6%)	**<0.001**	0.649
Dyslipidemia, n (%)	955 (47.8%)	315 (29.6%)	163 (92.1%)	238 (47.1%)	239 (95.6%)	**<0.001**	1.073
Proteinuria, n (%)	171 (8.1%)	55 (4.8%)	34 (19.0%)	34 (6.5%)	48 (19.2%)	**<0.001**	0.292
Antihypertensive Drug, n (%)	161 (7.7%)	36 (3.1%)	37 (20.7%)	25 (4.8%)	63 (25.2%)	**<0.001**	0.418
Antidiabetic Drug, n (%)	36 (1.7%)	5 (0.4%)	15 (8.4%)	3 (0.6%)	13 (5.2%)	**<0.001**	0.249
Dyslipidemia Drug, n (%)	33 (1.6%)	7 (0.6%)	4 (2.2%)	4 (0.8%)	18 (7.2%)	**<0.001**	0.199
Core Indices							
IRD-PS	-0.18 (-0.94, 0.72)	-0.64 (-1.23, 0.00)	1.18 (0.45, 2.08)	-0.04 (-0.64, 0.57)	1.35 (0.67, 2.29)	**<0.001**	1.001
SLI-PS	-0.16 (-1.02, 0.79)	-0.40 (-1.20, 0.60)	-0.01 (-0.80, 0.76)	0.03 (-0.84, 0.88)	0.35 (-0.56, 1.30)	**<0.001**	0.241
Outcomes							
CKM Stage (3-level), n (%)						**<0.001**	0.753
Early (Stage 0-1)	608 (29.0%)	472 (41.1%)	0 (0.0%)	136 (26.1%)	0 (0.0%)		
Mid (Stage 2)	646 (30.8%)	280 (24.4%)	71 (39.7%)	199 (38.1%)	96 (38.4%)		
Late (Stage 3-4)	846 (40.3%)	397 (34.6%)	108 (60.3%)	187 (35.8%)	154 (61.6%)		

Data are presented as median (interquartile range) for continuous variables and n (%) for categorical variables. *P* values were derived from the Kruskal-Wallis test for continuous variables and the chi-square test or Fisher’s exact test for categorical variables. SMD, Standardized Mean Difference; an SMD ≥ 0.2 indicates a meaningful imbalance. ABI, Ankle-Brachial Index; baPWV, Brachial-Ankle Pulse Wave Velocity; BMI, Body Mass Index; CKM, Cardiovascular-Kidney-Metabolic Syndrome; Cr, Creatinine; DBP, Diastolic Blood Pressure; EF, Ejection Fraction; eGFR, estimated Glomerular Filtration Rate; HbA1c, Glycated Hemoglobin A1c; HDL-C, High-Density Lipoprotein Cholesterol; IRD-PS, Insulin Resistance/Dyslipidemia Pathological Score; LDL-C, Low-Density Lipoprotein Cholesterol; LVMI, Left Ventricular Mass Index; MHO, Metabolically Healthy Obese; MHNO, Metabolically Healthy Non-Obese; MUO, Metabolically Unhealthy Obese; MUNO, Metabolically Unhealthy Non-Obese; SBP, Systolic Blood Pressure; SLI-PS, Systemic Low-grade Inflammation Pathological Score; TC, Total Cholesterol; TG, Triglycerides; UA, Uric Acid; UACR, Urinary Albumin-to-Creatinine Ratio; VA, Visceral Adiposity; WBC, White Blood Cell Count.

Bold values indicate statistical significance at P < 0.05.

**Figure 1:**
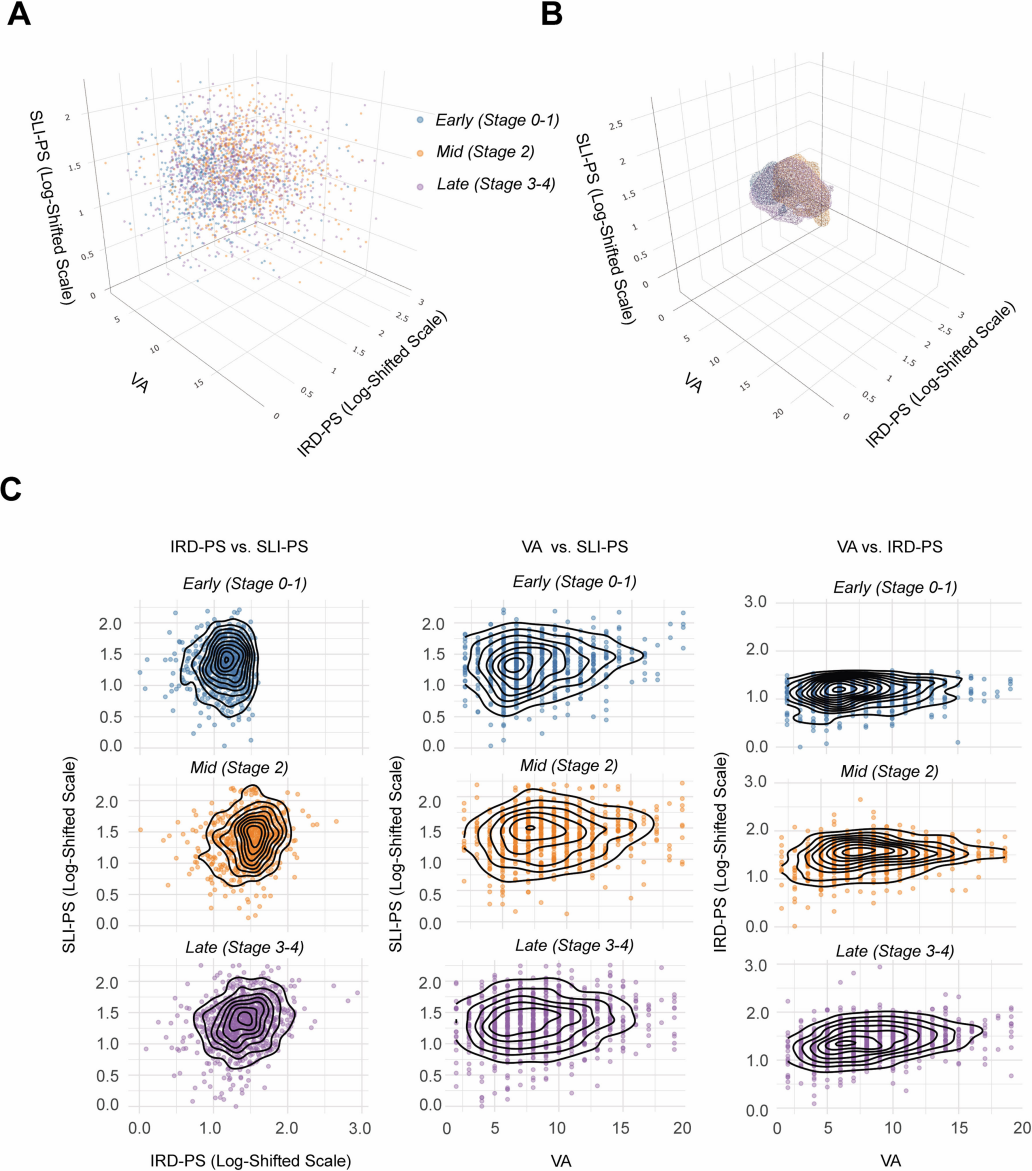
3D and 2D distributions of CKM stage in the pathophysiological space in Shaanxi sample (n = 2, 100). **(A)** 3D scatter plot showing the distribution of participants across the VA (Visceral Adiposity) axis, IRD-PS (Insulin Resistance/Dyslipidemia Pathological Score, log-shifted), and SLI-PS (Systemic Low-grade Inflammation Pathological Score, log-shifted). Points are color-coded by CKM stage: Early (Stage 0-1) (blue), Mid (Stage 2) (orange), and Late (Stage 3-4) (purple). **(B)** 3D kernel density isosurfaces (top 30% density) for each CKM stage, highlighting core spatial clusters in the pathological space. **(C)** Pairwise 2D scatter plots with semi-transparent points and overlaid kernel density contour lines, stratified by CKM stage.

Within both populations, three pathological axes were constructed: visceral adiposity (VA), an insulin resistance/dyslipidemia pathological score (IRD-PS), and a systemic low-grade inflammation pathological score (SLI-PS) (**Supplementary Table 3**). Intercorrelations among the axes were generally low (most |*ρ*| < 0.3), although a moderate correlation was noted between VA and IRD-PS in the NHANES sample (Pearson’s *ρ* = 0.45). Across the CKM spectrum, Early CKM syndrome (Stages 0–1) clustered at lower scores across all axes, Mid (Stage 2) showed intermediate scores, and Late (Stages 3–4) shifted toward higher VA, IRD-PS, and SLI-PS, illustrating a pattern of increasing pathological burden across the cardiovascular–kidney–metabolic (CKM) spectrum (**Figure 1**; **Supplementary Tables 5**, **S6**).

### Differential patterns of association with CKM status

Despite the notable differences in population characteristics and measurement protocols between the Shaanxi and NHANES samples, a consistent stage-specific pattern of association emerged in both samples.

Firth’s penalized logistic regression, justified by violations of the proportional odds assumption, revealed associations with established CKM status (Mid and Late [Stages 2–4] vs. Early [Stages 0–1]) and advanced CKM status (Late [Stages 3–4] vs. Early and Mid [Stages 0–2]) (**Table 2**). With respect to established CKM status, IRD-PS showed the strongest association in both the Shaanxi sample (fully adjusted *OR* = 2.49; 95% *CI*, 2.18, 2.86) and the NHANES sample (fully adjusted *OR* = 2.52; 95% *CI*, 2.24, 2.82). Conversely, for advanced CKM status, the SLI-PS was consistently associated in both the Shaanxi sample (fully adjusted *OR* = 1.11; 95% *CI*, 1.03, 1.19) and the NHANES sample (fully adjusted *OR* = 1.07; 95% *CI*, 1.01, 1.14).

**Table 2:** Stage-specific associations between pathological axes with CKM status.

Variables	Shaanxi Dataset (n = 2, 100)		NHANES (n = 5, 359)	
	OR (95% CI)	*P* value	OR (95% CI)	*P* value
Established CKM Status				
VA	1.05 (1.01, 1.09)	**0.006**	1.96 (1.54, 2.50)	**<0.001**
IRD-PS	2.49 (2.18, 2.86)	**<0.001**	2.52 (2.24, 2.82)	**<0.001**
SLI -PS	1.08 (0.99, 1.18)	0.073	1.06 (1.02, 1.11)	**0.008**
Advanced CKM Status				
VA	1.03 (1.00, 1.06)	**0.040**	1.37 (0.79, 2.38)	0.251
IRD-PS	1.02 (0.95, 1.10)	0.600	1.07 (0.99, 1.15)	0.070
SLI -PS	1.11 (1.03, 1.19)	**0.009**	1.07 (1.01, 1.14)	**0.028**

Firth penalization was applied to address data separation. Established CKM status: Mid (Stage 2)/Late (Stage 3-4) vs. Early (Stage 0-1). Advanced CKM status: Late (Stage 3-4) vs. Early (Stage 0-1)/Mid (Stage 2).

For Shaanxi data: The fully adjusted model included age, sex, smoking status, drinking status, physical activity, education level, family history of cardiovascular disease and family history of stroke.

For NHANES data: The fully adjusted model included age, sex, smoking status, drinking status, physical activity, education level and race/ethnicity. CI, Confidence Interval; CKM, Cardiovascular-Kidney-Metabolic; IRD-PS, Insulin Resistance/Dyslipidemia Pathological Score; OR, Odds Ratio; SLI-PS, Systemic Low-grade Inflammation Pathological Score; VA, Visceral Adiposity.

Bold values indicate statistical significance at P < 0.05.

However, population-specific divergences were identified (**Table 2**). The association of VA with advanced CKM status was modest but significant in the Shaanxi sample (fully adjusted *OR* = 1.03; 95% *CI*, 1.00, 1.06) but not in the NHANES sample (*P* = 0.251). In contrast, SLI-PS was also modestly associated with established CKM status in the NHANES sample (*OR* = 1.06; 95% *CI*, 1.02, 1.11) but not in the Shaanxi sample (*P* = 0.073).

For the Shaanxi sample, the IPW sensitivity analysis for Firth’s penalized logistic regression confirmed the robustness of these core associations: the weighted ORs for IRD-PS with established CKM status and SLI-PS with advanced CKM status were highly concordant with the primary CCA results (**Supplementary Table 7**). The IPW-weighted sub-sample also achieved excellent baseline balance with the overall Shaanxi survey population (SMDs < 0.1 for all demographic factors; **Supplementary Table 8**).

### Interplay of pathological axes: dose–response, exploratory decomposition, and synergism

**Figure 2:**
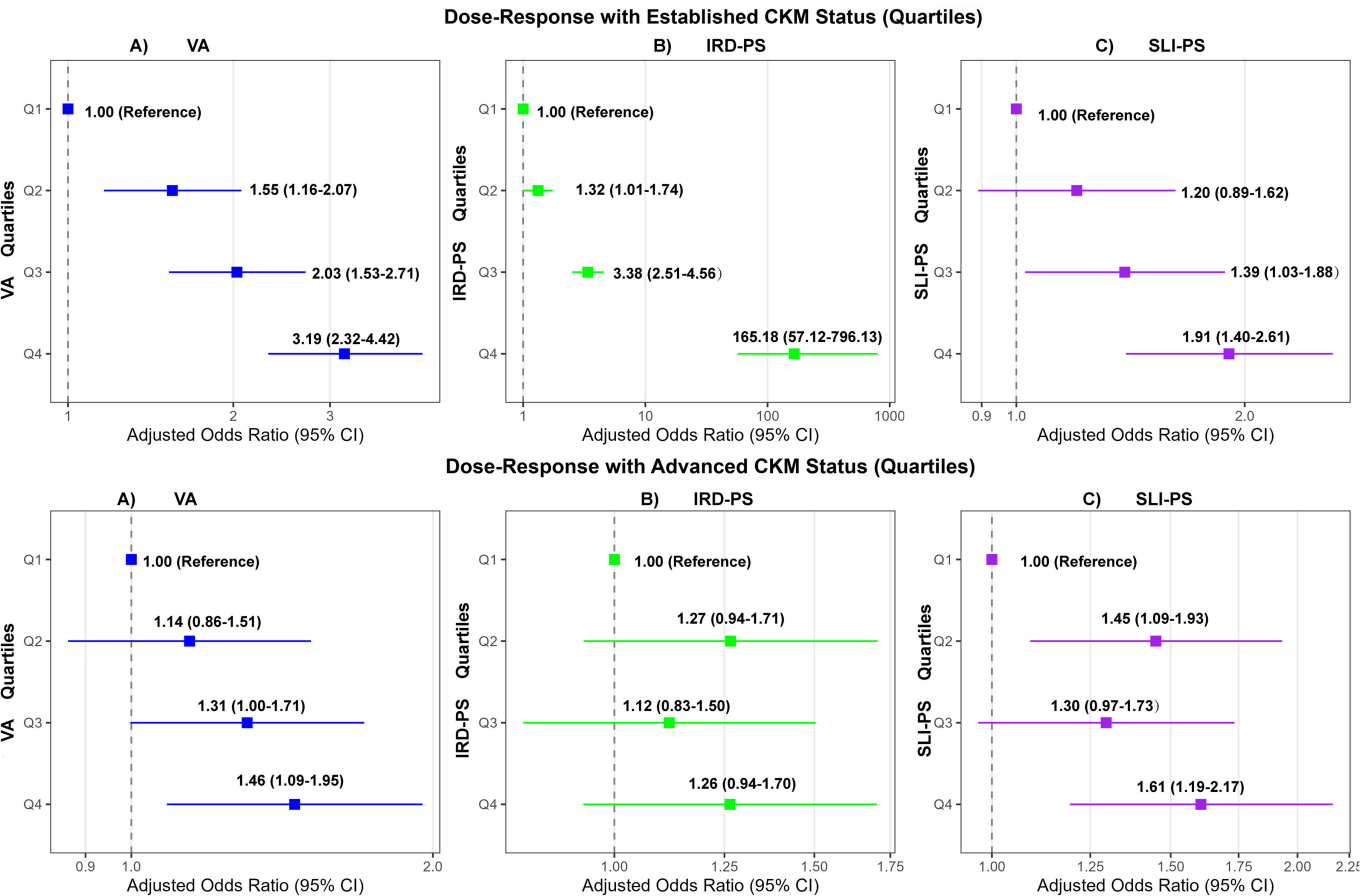
Dose-response relationship of pathological axes with established and advanced CKM status in the Shaanxi dataset (n = 2,100). Forest plots display the adjusted odds ratios (ORs) and 95% confidence intervals for established CKM status (top row, panels **A–C**) and advanced CKM status (bottom row, panels **A–C**). The associations are shown across quartiles (Q1–Q4) of **(A)** Visceral Adiposity (VA), **(B)** Insulin Resistance/Dyslipidemia Pathological Score (IRD-PS), and **(C)** Systemic Low-grade Inflammation Pathological Score (SLI-PS). The lowest quartile (Q1) serves as the reference group. Odds ratios are fully adjusted. Analyses were fully adjusted for age, sex, smoking status, drinking status, physical activity, education level, family history of cardiovascular disease, and family history of stroke.

Quartile-based analyses of the Shaanxi sample visually confirmed these patterns and revealed strong dose–response relationships. For established CKM status, a steep, graded relationship was observed for the IRD-PS; compared with the lowest quartile (Q1), the adjusted *OR* for participants in the highest quartile (Q4) was exceptionally high at 165.18 (95% *CI*, 57.12, 796.13) (**Figure 2**). This extreme magnitude of association is statistically justified by the Firth’s penalized regression, which addresses the quasi-complete separation observed in the raw data: nearly all participants (99.6%) in the highest IRD-PS quartile (Q4) were in the Mid or Late CKM stage, in stark contrast to 47.2% in the lowest quartile (Q1) (**Supplementary Table 9**). Of note, this extreme OR reflects the strong statistical association between severe metabolic dysregulation and established CKM status in this community-based dataset, and should be interpreted with caution for clinical generalization, as it is derived from quartile-based extreme group comparison. This association pattern remained robust in the sensitivity analysis (excluding dyslipidemia-driven Stage 2 cases), where the odds for the highest quartile of IRD-PS remained markedly higher (*OR* = 28.53; 95% *CI*, 13.90, 67.73; *P* < 0.001). With respect to advanced CKM status, the SLI-PS demonstrated a clear dose–response relationship (Q4 vs. Q1 *OR* = 1.61; 95% *CI*, 1.19, 2.17), whereas the association for the IRD-PS was null across all quartiles (*P* for trend = 0.236).

**Figure 3:**
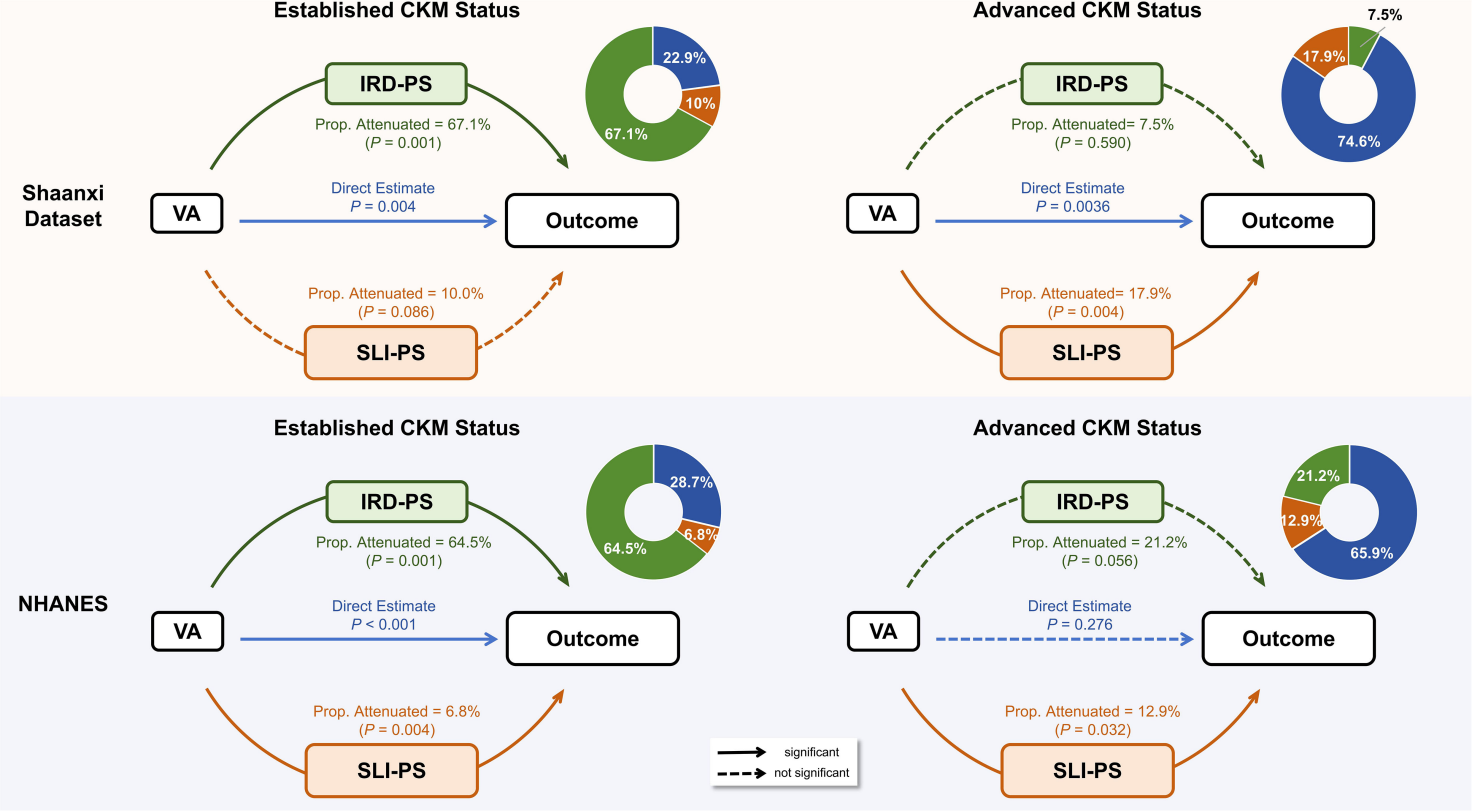
Exploratory decomposition analysis of IRD-PS and SLI-PS on the association between VA and CKM status. The diagrams illustrate the proportion of the association between Visceral Adiposity (VA) and CKM outcomes that is statistically attenuated by the Insulin Resistance/Dyslipidemia Pathological Score (IRD-PS) and the Systemic Low-grade Inflammation Pathological Score (SLI-PS). Analyses are presented for established CKM status (left) and advanced CKM status (right) in both the Shaanxi (n = 2, 100; top) and NHANES (n = 5, 359; bottom) datasets. The green path represents statistical attenuation by IRD-PS, and the orange path represents statistical attenuation by SLI-PS. Solid arrows indicate statistically significant associations (*P* < 0.05), while dashed arrows indicate non-significant associations. The donut charts summarize the proportion of the total association statistically attenuated by each axis. All underlying models were fully adjusted for age, sex, smoking status, drinking status, physical activity, education level, and dataset-specific covariates (family history of cardiovascular disease and stroke for Shaanxi; race/ethnicity for NHANES).

Exploratory decomposition analyses assessed the interrelationships of these axes (**Figure 3**). With respect to established CKM status, the association of VA was statistically attenuated by IRD-PS in both the Shaanxi (proportion statistically attenuated, 67.1%) and NHANES (64.5%) populations. However, for advanced CKM status, this relationship was no longer significant; instead, a smaller proportion of the association with VA was statistically attenuated by SLI-PS in both samples (Shaanxi: 17.9%; NHANES: 12.9%). A significant direct association of VA with CKM status remained in most models, suggesting the presence of other contributing pathways (details in **Supplementary Tables 10**, **S11**).

**Figure 4:**
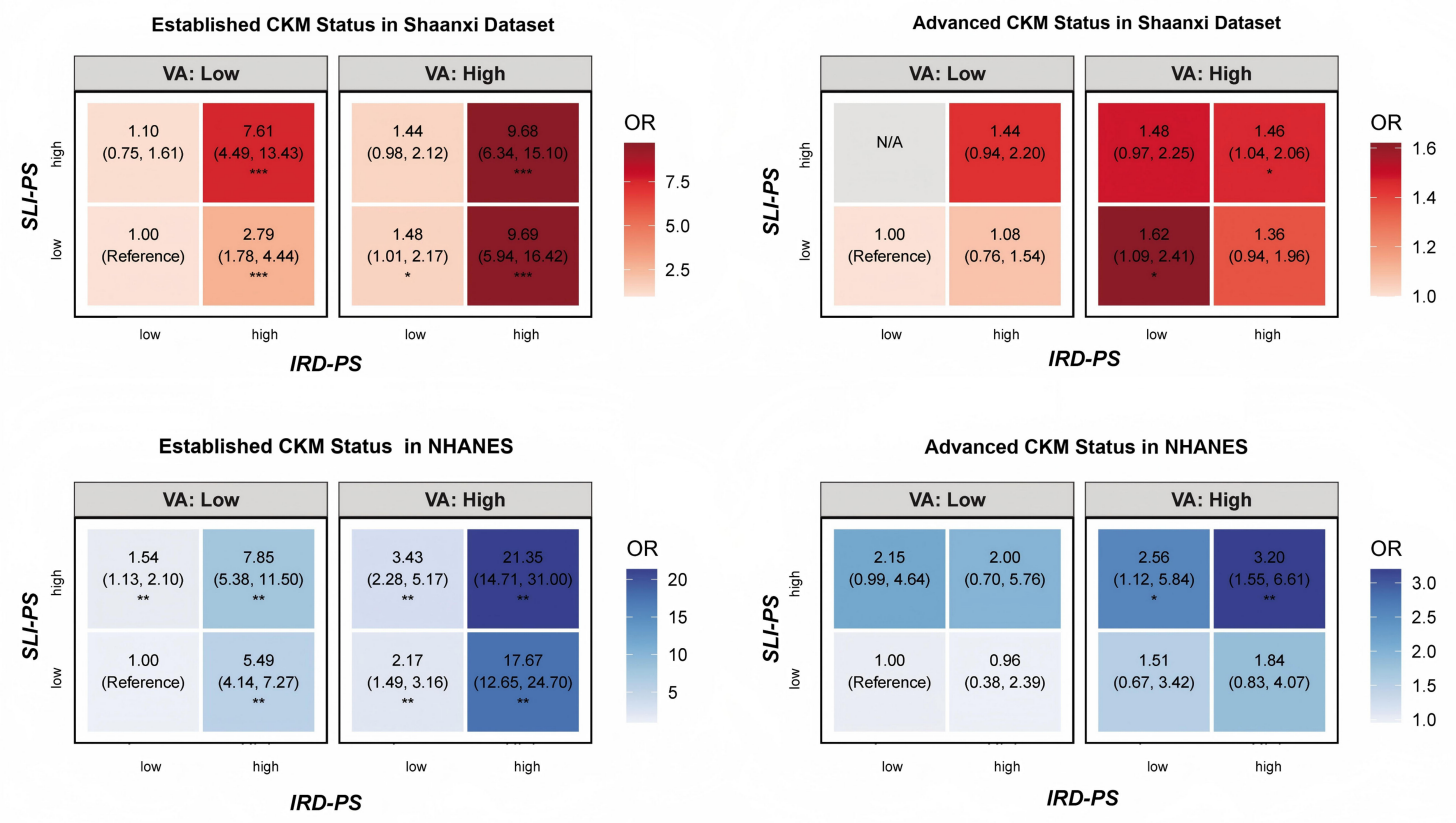
Synergistic associations of pathological axes with established and advanced CKM status. The heatmaps show the odds ratios (ORs) for the joint effects of Visceral Adiposity (VA), Insulin Resistance/Dyslipidemia Pathological Score (IRD-PS), and Systemic Low-grade Inflammation Pathological Score (SLI-PS) on CKM status. The analysis is stratified by established versus advanced CKM status and by dataset (Shaanxi, n = 2, 100; NHANES, n = 5, 359). Each pathological axis was dichotomized at its median value (Low vs. High). The reference group for all comparisons is the category with low levels on all respective axes. ORs are fully adjusted (covariates included age, sex, smoking status, drinking status, physical activity, education level, and dataset-specific variables: family history of cardiovascular disease/stroke for Shaanxi, and race/ethnicity for NHANES), and asterisks indicate the level of statistical significance.

Beyond individual and exploratory decomposition results, an analysis of combined exposures revealed strong synergistic associations with established CKM status in both populations (**Figure 4**). In the Shaanxi sample, the *OR* for participants with both high VA and high IRD-PS was 9.69 (95% *CI*, 5.94, 16.42). This synergistic pattern appeared even more pronounced in the NHANES sample, where the corresponding *OR* was 17.67 (95% *CI*, 12.65–24.70). For advanced CKM status in the NHANES sample, the highest risk was observed in the group with high values for all three axes (*OR* = 3.20; 95% *CI*, 1.55, 6.61), with the combination of high VA and high SLI-PS also showing a significant association (*OR* = 2.56; 95% *CI*, 1.12, 5.84).

### Associations with organ-specific outcomes

In the Shaanxi sample, the pathological axes were linked to distinct patterns of end-organ damage (**Table 3**). Specifically, VA was associated with arterial stiffness (*OR* = 1.06; 95% *CI*, 1.01, 1.12) and albuminuria (*OR* = 1.06; 95% *CI*, 1.01, 1.11). The IRD-PS was significantly associated with albuminuria (*OR* = 1.17; 95% *CI*, 1.05, 1.30). The SLI-PS demonstrated the broadest range of associations, linked to both cardiac dysfunction (*OR* = 1.09; 95% *CI*, 1.01, 1.17) and albuminuria (*OR* = 1.24; 95% *CI*, 1.11, 1.39).

**Table 3:** Associations of pathophysiological axes with organ-specific outcomes in Shaanxi dataset (n = 2, 100).

Outcome	VA		IRD-PS		SLI-PS	
	OR (95% CI)	*P* value	OR (95% CI)	*P* value	OR (95% CI)	*P* value
Arterial stiffness	1.06 (1.01, 1.12)	**0.028**	1.08 (0.95, 1.22)	0.218	1.12 (0.97, 1.28)	0.121
Cardiac dysfunction	1.03 (1.00, 1.07)	**0.037**	1.02 (0.95, 1.10)	0.583	1.09 (1.01, 1.17)	**0.026**
Albuminuria	1.06 (1.01, 1.11)	**0.011**	1.17 (1.05, 1.30)	**0.004**	1.24 (1.11, 1.39)	**<0.001**

Odds ratios (OR) are derived from multivariable logistic regression models adjusted for age, sex, smoking status, drinking status, physical activity, education level, family history of cardiovascular disease and family history of stroke. CI, Confidence Interval; CKM, Cardiovascular-Kidney-Metabolic; IRD-PS, Insulin Resistance/Dyslipidemia Pathological Score; OR, Odds ratios; SLI-PS, Systemic Low-Grade Inflammation Pathological Score; VA, Visceral Adiposity.

Bold values indicate statistical significance at P < 0.05.

### Heterogeneity in subgroup analyses

Finally, subgroup analyses revealed consistent patterns of effect modification and population-specific heterogeneity. First, our primary finding for established CKM status proved highly robust. The strong association between IRD-PS and established CKM status remained significant across sex and most age strata in both the Shaanxi and the NHANES populations. In contrast, the association between SLI-PS and advanced CKM status was consistently observed in specific subgroups: it was significant in women (Shaanxi *OR* = 1.13; 95% *CI*, 1.02, 1.25; NHANES *OR* = 1.09; 95% *CI*, 1.02, 1.18) and older adults (Shaanxi > 55 years *OR* = 1.40; 95% *CI*, 1.14, 1.75; NHANES > 50 years *OR* = 1.10; 95% *CI*, 1.01, 1.20) but nonsignificant in men and younger age groups in both samples (**Supplementary Tables 12**, **S13****;****Supplementary Figures 2**, **S3**).

Furthermore, subgroup exploratory decomposition analyses highlighted a key age-related divergence in the upstream correlates of established CKM status: the proportion of VA associations statistically attenuated by the IRD-PS decreased sharply with age in the Shaanxi sample (from 93.5% in the youngest quartile to 23.0% in the oldest quartile), a trend not observed in the NHANES sample where the high proportion of attenuation remained stable across all age groups (54.3–68.5%). Finally, population-specific findings emerged in the NHANES sample, where there was significant heterogeneity by race/ethnicity for the IRD-PS association: the odds ratio was strongest among non-Hispanic Asian people (*OR* = 3.96; 95% *CI*, 3.14, 5.00) and weakest among non-Hispanic Black people (*OR* = 2.03; 95% *CI*, 1.73, 2.39; *P* for interaction < 0.001) (**Supplementary Tables 14**, **S15****;****Supplementary Figures 4**, **S5**). Notably, this observed racial/ethnic heterogeneity represents an associative pattern only and does not imply biological determinism or causality.

## Discussion

The increasing global burden of cardiovascular-kidney-metabolic (CKM) syndrome calls for a deeper understanding of its pathophysiology to guide precision interventions (23). In this cross-sectional study of two distinct populations, the Shaanxi dataset (n = 2, 100) and the multiethnic NHANES (n = 5, 359), we identified statistical patterns consistent with a hypothetical stage-specific framework of CKM status. By quantifying three distinct pathological axes, namely, visceral adiposity (VA), insulin resistance/dyslipidemia pathological score (IRD-PS), and systemic low-grade inflammation pathological score (SLI-PS), we observed distinct associations with established versus advanced CKM status. Our primary finding was that the IRD-PS was most strongly associated with established CKM status (Shaanxi: *OR* = 2.49; 95% *CI*, 2.18, 2.86, *P* < 0.001; NHANES: *OR* = 2.52; 95% *CI*, 2.24, 2.82, *P* < 0.001), whereas the SLI-PS (Shaanxi: *OR* = 1.11; 95% *CI*, 1.03, 1.19, *P* = 0.009; NHANES: *OR* = 1.07; 95% *CI*, 1.01, 1.14, *P* = 0.028) was significantly associated with advanced CKM status. The stability of this framework across diverse populations and measurement techniques supports a hypothesized difference in correlational patterns. We observed that metabolic dysregulation was the dominant correlate of established CKM status, while an inflammation-predominant state was more prominent in advanced CKM stages. This pattern generates hypotheses for stage-specific interventions.

The strong association between the IRD-PS and established CKM status underscores the central role of early metabolic dysregulation. This aligns with established knowledge linking insulin resistance and dyslipidemia to cardiovascular risk, potentially through processes such as endothelial dysfunction and the formation of atherogenic lipid profiles (24). The sheer magnitude of this association was striking: in quartile-based analyses, participants with the highest IRD-PS levels had exceptionally elevated odds of being in an established CKM status (Shaanxi: Q4 vs. Q1 *OR* = 165.18, 95% *CI*, 57.12, 796.13, *P* < 0.001). This exceptionally high OR, while possessing a wide confidence interval, is a stable estimate from the Firth’s penalized regression necessitated by the quasi-complete data separation (**Supplementary Table 7**). An odds ratio of this magnitude suggests that at these high levels, severe metabolic dysregulation may be a defining characteristic of the established CKM state itself, perhaps reflecting a biological tipping point where compensatory mechanisms are overwhelmed (25, 26). Although the potential circularity in the primary analysis (due to the lipid components of the IRD-PS overlap with the dyslipidemia criteria for CKM Stage 2) remains an inherent limitation, our sensitivity analysis demonstrated that the strong association persists even when restricting the outcome to non-lipid-driven cases. These findings support the utility of composite biomarkers such as the TyG index in early screening protocols, which have proven effective in Asian populations, such as predicting prediabetes risk with an AUC of 0.73 in Chinese cohorts (27).

In contrast, the associations with the SLI-PS and VA were more pronounced for advanced CKM status. The link between SLI-PS and outcomes such as cardiac dysfunction (Shaanxi: *OR* = 1.09; *P* = 0.026) and albuminuria (Shaanxi: *OR* = 1.24; *P* < 0.001) suggests that it may be related to widespread systemic processes, such as cytokine-driven endothelial damage associated with the secretion of IL-1β, IL-6, and TNF-α from stimulated endothelial cells (4). Similarly, the associations of VA with arterial stiffness (Shaanxi: *OR* = 1.06; *P* = 0.028) and albuminuria (Shaanxi: *OR* = 1.06; *P* = 0.011) may reflect pathways such as renin angiotensin system activation, where a high visceral fat area is independently associated with an increased risk of albuminuria (*OR* = 1.37) in a population including patients with diabetes (28). The observation that the strong IRD-PS association disappears when analyzing advanced CKM status (Shaanxi: *P* = 0.236; NHANES: *P* = 0.070) further supports a conceptual difference in the primary pathological correlates in advanced CKM status, potentially as chronic metabolic stress gives way to sustained inflammatory amplification.

An important finding is the consistent pattern of associations observed across the two samples, despite significant methodological heterogeneity. While these differences, such as the use of BIA versus DXA for visceral fat assessment and whether inflammation was gauged with or without lymphocyte-based ratios, represent key limitations and prevent direct comparisons of absolute parameter estimates, the consistent pattern suggests that the observed associations are not merely artifacts of a single measurement technique. The hypothesized shift from a dominant metabolic association in established CKM status to an inflammatory association in advanced CKM status was evident. This suggests that the observed pattern reflects a robust pathophysiological signal, and the apparent robustness of this framework warrants its further investigation for risk stratification in diverse health care settings.

Our cross-sectional exploratory decomposition models suggest that VA is strongly correlated with both IRD-PS and SLI-PS. With respect to established CKM status, a substantial proportion of the association between VA and CKM status was statistically attenuated after adjusting for IRD-PS in both populations (Shaanxi: 67.1%, *Attenuation Estimate* = 0.0154, *P* < 0.001; NHANES: 64.5%, *Attenuation Estimate* = 0.0045, *P* < 0.001), which is consistent with the biological role of visceral fat in promoting hepatic insulin resistance and dyslipidemia via free fatty acid spillover and altered mTOR signaling (29–31). With respect to advanced CKM status, the statistical pattern was different, with the SLI-PS attenuating a smaller but significant proportion of the association with VA (Shaanxi: 17.9%, *Attenuation Estimate* = 0.0012, *P* = 0.004; NHANES: 12.9%, *Attenuation Estimate* = 0.0009, *P* = 0.032). The persistence of a direct association for VA in most models, for example, the Shaanxi direct effect (*β* = 0.0022, *P* < 0.001) for established CKM status, suggests that other pathways not captured by IRD-PS or SLI-PS, such as leptin resistance or extracellular vesicle-mediated signaling, are also associated with these outcomes, warranting further mechanistic studies (32, 33).

Furthermore, the synergistic interplay between these axes is associated with amplified risk. The combination of high VA and high IRD-PS was associated with markedly elevated odds of established CKM status (Shaanxi: *OR* = 9.69; 95% *CI*, 5.94, 16.42; NHANES: *OR* = 17.67; 95% *CI*, 12.65, 24.70), an effect particularly strong in the NHANES sample, possibly due to greater racial diversity amplifying metabolic adiposity interactions. This suggests that while metabolic dysregulation is a potent factor on its own, its conjunction with excess visceral adiposity is associated with a particularly high-risk state, exceeding expected additive odds by 2- to 3-fold in joint models.

Analysis of the multiethnic NHANES sample alongside the Chinese sample revealed both the robustness of the overall framework and important population-specific patterns of heterogeneity. For example, the association between IRD-PS and established CKM status, while universally strong, varied significantly by race/ethnicity (*P* for interaction < 0.001); it was the strongest in non-Hispanic Asian people (*OR* = 3.96; 95% *CI*, 3.14, 5.00) and the weakest in non-Hispanic Black people (*OR* = 2.03; 95% *CI*, 1.73, 2.39). Notably, this heterogeneity analysis was conducted within the NHANES population, where measurement methods were standardized across all racial/ethnic groups. Crucially, this observed racial/ethnic heterogeneity is an associative pattern and does not indicate biological determinism; the underlying differences are more likely driven by a combination of non-biological factors including socioeconomic status, structural inequities, disparities in healthcare access and utilization, cultural lifestyle and dietary patterns, and environmental exposures rather than inherent biological differences between racial/ethnic populations. While genetic predispositions (e.g., higher APOE ϵ4 allele frequency in Asian people) may modulate metabolic risk, these genetic factors interact with social and environmental determinants, which are associated with the observed heterogeneity. Asian people showing a 41.2% prevalence of MetS versus 26.7% in Black people, for instance, is closely linked to these modifiable non-biological factors (34, 35). Such findings underscore that although the broad stage-specific pattern may be generalizable, the specific weighting and interplay of risk factors can differ across populations, highlighting the need to move beyond a one-size-fits-all approach in global CKM prevention, particularly as urbanization in Asia may exacerbate these disparities (36, 37). However, a direct statistical comparison of the odds ratios between the Shaanxi and NHANES datasets is not appropriate because of the methodological differences, precluding conclusions about the relative magnitude of risk factors between the Chinese and U.S. populations.

The patterns observed in our study may help generate hypotheses for future clinical research. For example, our findings raise the hypothesis that individuals with a high IRD-PS are at higher risk for established CKM status. Whether such individuals would derive greater benefit from metabolically targeted interventions requires elucidation by future longitudinal studies and randomized controlled trials. Similarly, it could be hypothesized that therapies with anti-inflammatory effects may be important for reducing damage in later stages, but this remains speculative and requires formal investigation. Thus, our findings provide a basis to test whether early screening focused on IRD-PS could identify individuals at high risk for established CKM status. This generates the testable hypothesis that the relative benefit of therapies may differ by CKM stage. For example, while SGLT2 inhibitors, which reduced CKD progression by 39% in trials (38), have broad benefits, the notion that their maximal efficacy might be in early CKM stages is strictly speculative based on our cross-sectional data. In contrast, the suggestion that therapies with pleiotropic effects, such as GLP-1 receptor agonists (i.e., semaglutide), which lowered major adverse cardiovascular events (MACE) by 20% in obesity trials (39), may be more impactful in advanced stages where inflammation and adiposity appear more prominent is also speculative. All therapeutic implications derived from this study require formal validation through future longitudinal cohort studies and prospective randomized controlled trials.

Our findings on sex- and age-specific heterogeneity, such as the stronger association of the SLI-PS with advanced CKM status in women (Shaanxi: *OR* = 1.13; 95% *CI*, 1.02, 1.25, *P* = 0.022; NHANES: *OR* = 1.09; 95% *CI*, 1.02, 1.18, *P* = 0.019) and older adults (Shaanxi >55 years: *OR* = 1.40; 95% *CI*, 1.14, 1.75, *P* = 0.001), further suggest that tailored strategies may be warranted, potentially addressing phenomena such as post-menopausal estrogen deprivation, which exacerbates immunosenescence and increases proinflammatory cytokines in women (40).

Beyond the U.S. and China, our metabolic-to-inflammatory framework is highly relevant to low- and middle-income countries (LMICs) facing a ‘double burden’ of malnutrition and rapid urbanization (41, 42). By 2035, nearly 79% of the world’s adults with overweight or obesity are projected to reside in LMICs, where limited healthcare resources may accelerate progression toward advanced, inflammatory-dominant CKM stages (43). The observed consistency across our diverse samples suggests these pathological drivers are fundamental biological processes that necessitate targeted validation in resource-limited settings.

### Strengths and limitations

The strengths of this study include its large sample size (total n = 7, 459), the use of PCA to create integrated pathological scores, and the comparative design across two distinct populations. We further strengthened the statistical robustness by combining complete-case primary analysis with IPW sensitivity analysis for the Shaanxi sample, and strictly following official CDC guidelines for NHANES survey weighting. A unique strength is the demonstrated robustness of our conceptual framework. We intentionally compared two distinct participant groups with inherent differences in measurement protocols (e.g., BIA vs. DXA; differing inflammatory biomarkers) to explore the generalizability of our framework. The core stage-specific pattern was consistent in the primary analysis (**Table 2**). Furthermore, our harmonization sensitivity analysis confirmed that this pattern remained robust even when constructing the axes using only identical, shared variables (**Supplementary Table 16**). This provides evidence that our findings are not merely an artifact of specific instruments but reflect a consistent pathophysiological pattern.

However, several limitations must be acknowledged. First, complete-case analysis was adopted for primary analyses. In the large NHANES dataset (N = 22, 617), while baseline differences were highly statistically significant, standardized mean differences (SMDs) revealed that core demographic factors (e.g., sex, race/ethnicity) and weight remained highly balanced (SMD < 0.1) between included and excluded participants (**Supplementary Table 2****).** The primary imbalance was observed in age (SMD = 0.860), reflecting non-random missingness inherent to rigorous NHANES examination protocols (excluding older adults from DXA scans). Although we implemented IPW to mitigate selection bias in the Shaanxi dataset, we acknowledge that our findings primarily generalize to the analyzed subsamples, and extrapolation to broader populations should be made cautiously. Second, the cross-sectional design precludes any inference of causality. The strict assumptions required for causal mediation analysis (e.g., temporal ordering) are unlikely to hold in cross-sectional data. Our exploratory decomposition findings should be interpreted strictly as statistical attenuation rather than true causal mediation. The observed associations provide a basis for hypothesis generation but require confirmation in longitudinal studies. Third, the measurement differences between the two samples, most notably the use of BIA-derived visceral fat grade in the Shaanxi sample versus DXA-derived visceral fat area in the NHANES, introduce systematic variability. While the two metrics are conceptually aligned to capture visceral adiposity, they are not directly equivalent, which precludes a direct comparison of the magnitude of VA associations between the two populations (44–46). Finally, despite extensive adjustment for confounders such as age, sex, and lifestyle, residual confounding from unmeasured factors, such as detailed dietary patterns including high glycemic loads or specific genetic variants like PPARγ polymorphisms, cannot be ruled out (47, 48).

## Conclusion

In this cross-sectional study, we observed a consistent pattern of pathological associations with CKM syndrome, suggesting that insulin resistance/dyslipidemia was the predominant correlate of established CKM status, whereas systemic inflammation was more prominent in advanced stages. Despite inherent differences in measurement protocols, the consistency of this framework across a Chinese population and a multiethnic U.S. population underscores its robustness. We acknowledge that these findings may only generalize to the analyzed subsamples rather than the broader Chinese or U.S. populations. Additionally, these core findings were complemented by important population-specific nuances, such as the stronger statistical attenuation of the visceral adiposity association in the Shaanxi sample (67.1%) and significant racial/ethnic variations in metabolic risk within the U.S. population. Together, these findings contribute to a conceptual framework that generates hypotheses for stage-specific risk stratification that may help guide more targeted and potentially more equitable strategies to address the growing global burden of CKM syndrome. Future longitudinal cohorts and randomized controlled trials are critical to validate our findings and translate this stage-specific framework into evidence-based clinical practice.

## Data Availability

The raw data supporting the conclusions of this article will be made available by the authors, without undue reservation.
