# Supplementary material for: A metabolic-to-inflammatory pattern in cardiovascular-kidney-metabolic syndrome staging: a comparative cross-sectional study
Source: Front Endocrinol (Lausanne). 2026 Apr 22;17:1805355. doi: 10.3389/fendo.2026.1805355 (PMC13143627; doi:10.3389/fendo.2026.1805355)
Supplement: Supplementary file 1 [file DataSheet1.docx]

**Supplementary Material: Table of Contents**

**Supplementary Methods**

**Supplementary Method S1.** Anthropometric Measurements Protocol

**Supplementary Method S2.** Hemodynamic Measurements Protocol

**Supplementary Method S3.** Cardiovascular Assessments Protocol

**Supplementary Method S4.** Derivation of Clinical and Laboratory Variables

**References**

**Supplementary Tables**

**Supplementary Table S1.** Baseline characteristics of the included analytical sample (n=2,100) versus excluded participants (n=7,236) in the Shaanxi dataset.

**Supplementary Table S2.** Baseline characteristics of the included analytical sample (n=5,359) versus excluded participants (n=17,258) in the NHANES dataset (2011–2018).

**Supplementary Table S3.** Principal Component Analysis Loadings, Eigenvalues, and Variance Explained for IRD-PS and SLI-PS (First Principal Component)

**Supplementary Table S4.** Baseline Characteristics of Participants by Metabolic Phenotype (Weighted NHANES Data).

**Supplementary Table S5.** Study Population Characteristics Stratified by CKM Stage in Shaanxi Dataset.

**Supplementary Table S6.** Baseline Characteristics of Participants by CKM Stage (Weighted NHANES Data).

**Supplementary Table S7.** Population Representativeness and Balance Check via Inverse Probability Weighting (IPW) in Shaanxi Sample

**Supplementary Table S8.** IPW-weighted Models for Pathological Axes and CKM Stages in Shaanxi Sample

**Supplementary Table S9.** Raw counts of participants by Quartiles of Pathological Axes and CKM Stage in Shaanxi Sample.

**Supplementary Table S10.** Exploratory Decomposition Analysis of the Association Between Visceral Adiposity and Stage-Specific CKM Risk in Shaanxi Sample.

**Supplementary Table S11.** Exploratory Decomposition Analysis of the Association Between Visceral Adiposity and Stage-Specific CKM Risk in the NHANES.

**Supplementary Table S12.** Subgroup Analysis for the Association of Pathological Axes With CKM Stage in Shaanxi Sample.

**Supplementary Table S13.** Subgroup Analysis for the Association of Pathological Axes With CKM Stage in the NHANES.

**Supplementary Table S14.** Exploratory Decomposition of VA Associations Through IRD-PS and SLI-PS in Shaanxi Sample.

**Supplementary Table S15.** Exploratory Decomposition of VA Associations Through IRD-PS and SLI-PS in the NHANES.

**Supplementary Table S16.** Stage-Specific Associations Between Pathological Axes with CKM Stage (Harmonization Sensitivity Analysis).

**Supplementary Figures**

**Supplementary Figure S1.** Study participant flow diagram illustrating inclusion and exclusion criteria for the Chinese Shaanxi dataset (N = 2,100) and NHANES (N = 5,393).

**Supplementary Figure S2.** Subgroup analysis of the associations between pathological axes and CKM stages, stratified by age and sex in the Shaanxi dataset.

**Supplementary Figure S3.** Subgroup analysis of the associations between pathological axes and CKM stages, stratified by age, sex, and race/ethnicity in the NHANES dataset.

**Supplementary Figure S4.** Waterfall plots of statistical attenuation proportions for the association of VA with CKM stages, stratified by sex and age in the Shaanxi dataset.

**Supplementary Figure S5.** Waterfall plots of statistical attenuation proportions for the association of VA with CKM stages, stratified by sex, age, and race/ethnicity in the NHANES dataset.

# **Supplementary Methods**

## **Supplementary Method S1. Anthropometric Measurements Protocol**

All anthropometric measurements were performed on participants wearing light indoor clothing after removing shoes, socks, and heavy outer garments.

*Height*

Preparation: Participants stood erect against a stadiometer (Model BT-24, Suhong, China), ensuring their heels, buttocks, and scapulae were in contact with the vertical board. The head was positioned in the Frankfurt horizontal plane (the ear tragus level with the inferior orbital margin).

Procedure: A movable headboard was lowered until it made firm contact with the crown of the head, without compressing the hair or scalp.

Recording and Repetition: Height was recorded to the nearest 0.1 cm. The procedure was repeated once. If the two measurements differed by less than 0.5 cm, their average was used for analysis. Otherwise, a third measurement was conducted.

*Waist Circumference (WC)*

Preparation: The measurement was performed directly on bare skin.

Procedure: A non-stretchable measuring tape was positioned horizontally around the abdomen at the midpoint between the inferior costal margin (lowest rib) and the superior iliac crest, aligned with the mid-axillary line.

Recording and Repetition: The circumference was measured at the end of a normal expiration, ensuring the tape was snug but did not compress the skin. The value was recorded to the nearest 0.1 cm. The measurement was repeated, and if the two readings differed by less than 0.5 cm, their average was used.

*Body Weight and Composition*

Pre-measurement Conditions: Participants were in a fasting state, had voided their bladder, and rested in a standing position for at least 5 minutes prior to the measurement.

Procedure: Body weight, body fat percentage, and visceral fat grade were measured using a multi-frequency bioelectrical impedance analysis (BIA) device (InBody 770, InBody Co., Ltd., Seoul, Korea)^1^. Participants stood barefoot on the device's electrodes and held the handgrip electrodes with arms extended at approximately a 15-degree angle, ensuring no contact with the torso.

Exclusion Criteria: Individuals with implanted electronic medical devices, such as pacemakers, were excluded from BIA measurements.

**Supplementary Method S2. Hemodynamic Measurements Protocol**

*Brachial Blood Pressure (BP)*

Pre-measurement Conditions: Measurements were taken in a quiet room after the participant had rested in a seated position for at least 5 minutes.

Procedure: An automated electronic sphygmomanometer (Omron HBP-1120U, Omron Healthcare, Kyoto, Japan) with an appropriately sized cuff was used. The cuff was placed on the participant's bare right upper arm, which was supported at heart level.

Measurement and Validation: Three consecutive measurements were taken at 1-minute intervals. The average of the three valid readings was used for analysis. If the systolic or diastolic pressure readings differed by more than 10 mmHg between any two measurements, the entire series was repeated.

*Ankle-Brachial Index (ABI) and Brachial-Ankle Pulse Wave Velocity (baPWV)*

Pre-measurement Conditions: Measurements were performed after the participant had rested in a supine position, without a pillow, for 10 to 15 minutes.

Procedure: An automated waveform analyzer (MB-3000, Maibang, China) was used. Cuffs were placed on both upper arms and ankles. Electrocardiogram (ECG) electrodes were attached to both wrists, and a phonocardiogram sensor was positioned over the sternum.

Data Acquisition: The device simultaneously measured brachial and ankle blood pressures to calculate the ABI and determined the pulse transit time to calculate the baPWV.

**Supplementary Method S3. Cardiovascular Assessments Protocol**

*Electrocardiogram (ECG)*

Procedure: A standard 12-lead resting ECG was recorded using a digital electrocardiograph (ECG-1212, Maibang, China) while the participant was in a relaxed, supine position. Electrodes were placed according to standard anatomical locations for limb and precordial (V1–V6) leads.

Data Recording: The complete 12-lead ECG waveform was recorded and stored digitally for analysis.

*Transthoracic Echocardiography*

Procedure: A comprehensive examination was conducted by a certified sonographer using a GE Vivid E9 ultrasound system.

Image Acquisition: Standard 2D, M-mode, and Doppler images were acquired according to established clinical guidelines.

Key Measurements: Key parameters for assessing cardiac structure and function were measured, including left ventricular dimensions, wall thickness, left ventricular ejection fraction (LVEF), and mitral inflow velocities (E and A peaks) for diastolic function assessment. All images were stored digitally for offline analysis.

**Supplementary Method S4. Derivation of Clinical and Laboratory Variables**

1. *Body Mass Index (BMI)*

$$BMI=\frac{Weight (kg)}{[Height (m)]^{2}}$$

Unit: $kg/m^{2}$

1. *Estimated Glomerular Filtration Rate (eGFR)*

Using 2021 Chronic Kidney Disease Epidemiology Collaboration (CKD-EPI) equation (without race adjustment)^2^

$$eGFR=141\times{\min\left( \frac{\mathrm{Scr}}{\kappa},1 \right)}^{\alpha}\times{\max\left( \frac{\mathrm{Scr}}{\kappa},1 \right)}^{-1.209}\times0.993^{\mathrm{age}}\times\left\{ \begin{matrix} 1.018 & (Female) \\ 1 & (Male) \end{matrix} \right.$$

Variables:

$\mathrm{Scr}$ : Serum creatinine (in $mg/dL$ )

$\kappa$ : 0.7 (female), 0.9 (male).

$\alpha$ : -0.329 (female), -0.411 (male).

Unit: $mL/\min/1.73 m^{2}$

1. *Urinary Albumin-to-Creatinine Ratio (UACR)*

$$\text{UACR=}\frac{\text{Urinary Albumin (mg/L)}}{\text{Urinary Creatinine (g/L)}}$$

Unit: mg/g

1. *Triglyceride-Glucose (TyG) Index*^3^

$$\text{TyG=}\ln\left( \frac{\text{Fasting Triglycerides (mg/dL)×Fasting Glucose (mg/dL)}}{2} \right)$$

2. *Chronic Kidney Disease (CKD) Risk Stratification*

Based on the Kidney Disease: Improving Global Outcomes (KDIGO) guidelines^4,5^, using eGFR (G stages) and UACR (A stages).

Risk levels were determined via the KDIGO heat map: low risk (green), moderately increased risk (yellow), high risk (orange), very high risk (red).


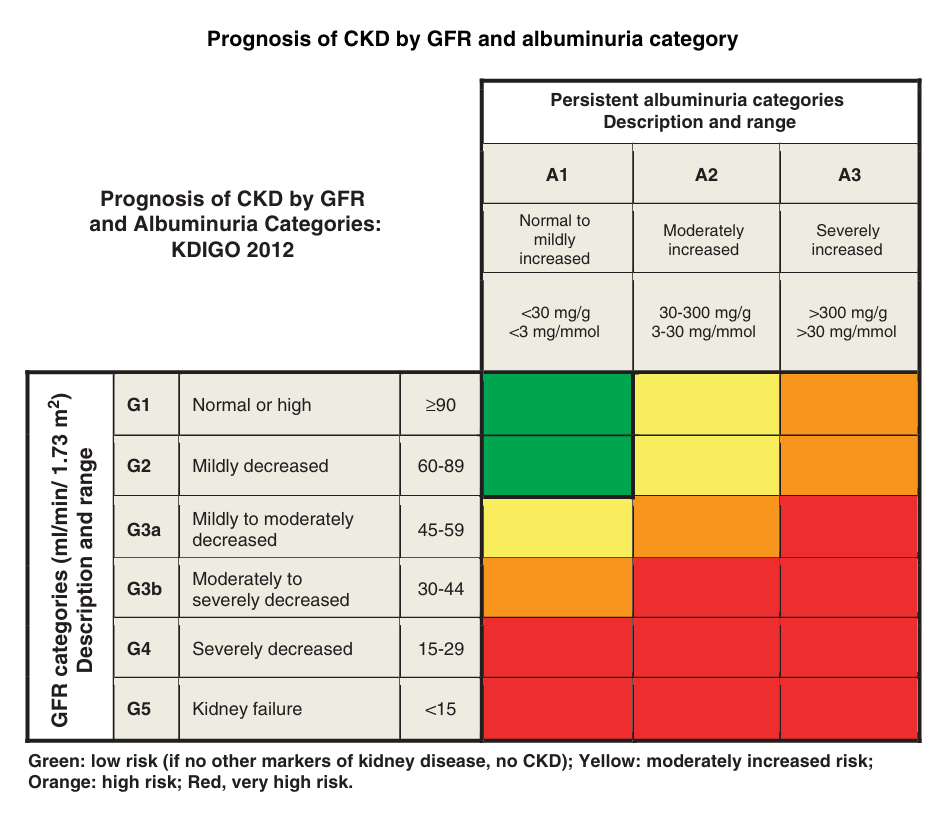


(Prognosis of CKD by GFR and Albuminuria Categories: KDIGO 2012[])

Staging Criteria:

| **eGFR Category**  **(G Stage)** | **eGFR**  **($\mathbf{mL/}\min\mathbf{/}\mathbf{1.73}\mathbf{m}^{\mathbf{2}}$)** | **UACR Category (A Stage)** | **UACR (mg/g)** |
| --- | --- | --- | --- |
| G1 | ≥ 90 | A1 | < 30 |
| G2 | 60–89 | A2 | 30–299 |
| G3a | 45–59 | A3 | ≥ 300 |
| G3b | 30–44 | - | - |
| G4 | 15–29 | - | - |
| G5 | < 15 | - | - |

Simplified CKD Staging (in Shaanxi dataset):

Normal: eGFR ≥ 90.

Subclinical: eGFR 60–89.

CKD: eGFR < 60.

Albuminuria: UACR ≥ 30 mg/g.

1. *Homeostatic Model Assessment for Insulin Resistance (HOMA-IR)*^6^

$$\text{HOMA−IR=}\frac{\text{Fasting Glucose (}\text{mmo/}\text{L)×Insulin (μU/mL)}}{22.5}$$

2. *Neutrophil-to-Lymphocyte Ratio (NLR)*

$$\text{NLR=}\frac{\text{Peripheral Blood Neutrophil Count (cells/}\text{μ}\text{L)}}{\text{Peripheral Blood Lymphocyte Count (cells/}\text{μ}\text{L)}}$$

3. *Platelet-to-Lymphocyte Ratio (PLR)*

$$\text{PLR=}\frac{\text{Peripheral Blood Platelet Count (×1}0^{9}\text{/L)}}{\text{Peripheral Blood Lymphocyte Count (×1}0^{9}\text{/L)}}$$

4. *Definition of Metabolic Syndrome (MetS) Components*

To ensure methodological consistency with the Shaanxi sample, metabolic phenotypes in the NHANES were defined using a modified 5-component definition of Metabolic Syndrome (MetS)^7^. The presence of three or more of the following five components, defined using U.S.-specific clinical thresholds, categorized a participant as “metabolically unhealthy”:

1) Elevated blood pressure: Defined as a mean systolic blood pressure ≥130 mmHg, mean diastolic blood pressure ≥85 mmHg, or a self-reported use of antihypertensive medication.

2) Hyperglycemia: Defined as a fasting plasma glucose ≥100 mg/dL, or a self-reported use of anti-diabetic medication.

3) Hypertriglyceridemia: Defined as fasting serum triglycerides (TG) ≥150 mg/dL.

4) Low HDL-C: Defined as a high-density lipoprotein cholesterol (HDL-C) level <40 mg/dL in men or <50 mg/dL in women.

5) Abdominal obesity: Defined as waist circumference ≥102 cm in men or ≥88 cm in women.

1. *Operationalization of CKM Staging*

CKM staging was adapted from the 2023 American Heart Association (AHA) Presidential Advisory, operationalized using variables available in the NHANES dataset as follows^8^:

Age, sex, race/ethnicity, and CVD history were ascertained via self-report; diabetes/hypertension were defined by biomarkers or clinical diagnoses. eGFR was calculated using the race-free CKD-EPI 2021 equation.

- Stage 0: Normal BMI (<25 kg/m²) and normal waist circumference (women <88 cm, men <102 cm), with no other CKM stage criteria met.
- Stage 1: Elevated BMI (>25 kg/m²), elevated waist circumference (women ≥88 cm, men ≥102 cm), or prediabetes (HbA1c 5.7%–6.5% or fasting glucose 100–126 mg/dL).
- Stage 2: Metabolic risk factors (hypertension, diabetes, metabolic syndrome, or elevated triglycerides ≥135 mg/dL) or moderate-to-high-risk CKD (per KDIGO criteria), as recommended by AHA.
- Stage 3: Very-high-risk KDIGO CKD or 10-year CVD risk ≥20% (estimated via AHA’s PREVENT equations; adults ≥80 years assigned age 79 for calculation).
- Stage 4: Self-reported established CVD (coronary heart disease, angina, myocardial infarction, heart failure, stroke); atrial fibrillation/peripheral artery disease excluded (no NHANES data).

**References:**

^1^ Malavolti, M. *et al.*, Cross-calibration of eight-polar bioelectrical impedance analysis versus dual-energy X-ray absorptiometry for the assessment of total and appendicular body composition in healthy subjects aged 21-82 years. *ANN HUM BIOL* **30** 380 (2009).

^2^ Inker, L. A. *et al.*, New Creatinine- and Cystatin C–Based Equations to Estimate GFR without Race. *NEW ENGL J MED* **385** 1737 (2021).

^3^ Simental-Mendía, L. E., Rodríguez-Morán, M. & Guerrero-Romero, F., The Product of Fasting Glucose and Triglycerides As Surrogate for Identifying Insulin Resistance in Apparently Healthy Subjects. *METAB SYNDR RELAT D* **6** 299 (2008).

^4^ Stevens, P. E. *et al.*, KDIGO 2024 Clinical Practice Guideline for the Evaluation and Management of Chronic Kidney Disease. *KIDNEY INT* **105** S117 (2024).

^5^ Stevens, P. E. & Levin, A., Evaluation and Management of Chronic Kidney Disease: Synopsis of the Kidney Disease: Improving Global Outcomes 2012 Clinical Practice Guideline. *ANN INTERN MED* **158** 825 (2013).

^6^ Matthews, D. R. *et al.*, Homeostasis model assessment: insulin resistance and ?-cell function from fasting plasma glucose and insulin concentrations in man. *DIABETOLOGIA* **28** 412 (1985).

^7^ Executive Summary of The Third Report of The National Cholesterol Education Program (NCEP) Expert Panel on Detection, Evaluation, And Treatment of High Blood Cholesterol In Adults (Adult Treatment Panel III). *JAMA-J AM MED ASSOC* **285** 2486 (2001).

^8^ Aggarwal, R., Ostrominski, J. W. & Vaduganathan, M., Prevalence of Cardiovascular-Kidney-Metabolic Syndrome Stages in US Adults, 2011-2020. *JAMA-J AM MED ASSOC* **331** 1858 (2024).

# **Supplementary Tables**

**Supplementary Table 1. Baseline characteristics of the included analytical sample (n=2,100) versus**

**excluded participants (n=7,236) in the Shaanxi dataset.**

| **Characteristic** | **Overall**  **(n = 9,336)** | **Included**  **(n = 2,100)** | **Excluded**  **(n = 7,236)** | ***P* value** |
| --- | --- | --- | --- | --- |
| Age (years) | 43 (31, 58) | 42 (31, 55) | 44 (31, 59) | <0.001 |
| Sex (Male), n (%) | 4,189 (45%) | 955 (45%) | 3,234 (45%) | >0.900 |
| Education Level, n (%) |  |  |  | <0.001 |
| Primary or below | 3,265 (35%) | 770 (37%) | 2,495 (34%) |  |
| Junior high graduation | 2,326 (25%) | 471 (22%) | 1,855 (26%) |  |
| Secondary graduate | 1,474 (16%) | 303 (14%) | 1,171 (16%) |  |
| Junior college and above | 2,271 (24%) | 556 (26%) | 1,715 (24%) |  |
| Lack of activity, n(%) | 1,093 (12%) | 346 (16%) | 747 (10%) | <0.001 |
| Current Smoker, n (%) | 1,624 (18%) | 296 (14%) | 1,328 (19%) | <0.001 |
| Drinker, n (%) | 1,542 (17%) | 392 (19%) | 1,150 (16%) | 0.006 |
| Height (cm) | 162 (156, 169) | 162 (158, 169) | 162 (156, 169) | <0.001 |
| BMI (kg/m²) | 23.9 (21.5, 26.5) | 22.9 (20.6, 25.3) | 24.2 (21.7, 26.8) | <0.001 |
| Waist (cm) | 85 (77, 94) | 80 (75, 88) | 87 (78, 96) | <0.001 |
| TC (mmol/L) | 4.40 (3.86, 5.06) | 4.27 (3.75, 4.87) | 4.45 (3.89, 5.12) | <0.001 |
| TG (mmol/L) | 1.29 (0.87, 2.00) | 1.25 (0.85, 1.97) | 1.30 (0.88, 2.00) | 0.035 |
| HDL-C (mmol/L) | 1.30 (1.12, 1.50) | 1.37 (1.21, 1.57) | 1.28 (1.10, 1.48) | <0.001 |
| LDL-C (mmol/L) | 2.40 (1.96, 2.93) | 2.42 (2.01, 2.92) | 2.40 (1.95, 2.93) | 0.200 |
| Glucose (mmol/L) | 4.97 (4.57, 5.54) | 4.94 (4.60, 5.44) | 4.97 (4.56, 5.57) | 0.300 |
| WBC (×10⁹/L) | 5.82 (4.92, 6.83) | 5.88 (4.95, 6.80) | 5.79 (4.91, 6.84) | 0.300 |
| SBP (mmHg) | 128 (115, 144) | 122 (111, 136) | 130 (116, 146) | <0.001 |
| DBP (mmHg) | 80 (73, 89) | 80 (72, 87) | 80 (73, 89) | 0.002 |

Note: Data are presented as median (interquartile range) for continuous variables and n (%) for categorical variables. *P* values were derived from the Wilcoxon rank-sum test for continuous variables and the Pearson’s chi-squared test or Fisher's exact test for categorical variables.

Abbreviations: BMI: Body Mass Index; DBP: Diastolic Blood Pressure; HDL-C: High-Density Lipoprotein Cholesterol; LDL-C: Low-Density Lipoprotein Cholesterol; SBP: Systolic Blood Pressure; TC: Total Cholesterol; TG: Triglycerides; WBC: White Blood Cell Count.

**Supplementary Table 2. Baseline characteristics of the included analytical sample (n=5,359) versus**

**excluded participants (n=17,258) in the NHANES dataset (2011–2018).**

| **Characteristic** | **Overall  (n = 22,617)** | **Included (n = 5,359)** | **Excluded  (n = 17,258)** | ***P* value** | **SMD** |
| --- | --- | --- | --- | --- | --- |
| Age (years) | 50 (34, 64) | 41 (30, 50) | 55 (37, 68) | <0.001 | 0.860 |
| Sex (Male), n (%) | 10,947 (48%) | 2,787 (52%) | 8,160 (48%) | <0.001 | 0.061 |
| Race/Ethnicity, n (%) |  |  |  | <0.001 | 0.077 |
| Mexican American | 3,037 (13%) | 755 (14%) | 2,282 (13%) |  |  |
| Non-Hispanic Asian people | 2,962 (13%) | 760 (14%) | 2,202 (13%) |  |  |
| Non-Hispanic Black people | 5,128 (23%) | 1,130 (21%) | 3,998 (23%) |  |  |
| Non-Hispanic White people | 8,311 (37%) | 1,926 (36%) | 6,385 (37%) |  |  |
| Other/Multi-Racial | 3,197 (14%) | 788 (15%) | 2,391 (14%) |  |  |
| Education Level, n (%) |  |  |  | <0.001 | 0.191 |
| Less than 9th grade | 2,172 (10%) | 209 (4%) | 1,963 (12%) |  |  |
| 9-11th grade | 2,887 (13%) | 531 (10%) | 2,356 (14%) |  |  |
| High school/GED | 5,033 (22%) | 1,179 (22%) | 3,854 (22%) |  |  |
| Some college/AA degree | 6,897 (30%) | 1,715 (32%) | 5,182 (30%) |  |  |
| College graduate or above | 5,598 (25%) | 1,725 (32%) | 3,873 (22%) |  |  |
| Marital Status, n (%) |  |  |  | <0.001 | 0.437 |
| Married/Living with partner | 13,198 (59%) | 3,376 (63%) | 9,822 (57%) |  |  |
| Divorced/Separated/Widowed | 5,046 (22%) | 697 (13%) | 4,349 (25%) |  |  |
| Never married | 4,354 (19%) | 1,286 (24%) | 3,068 (18%) |  |  |
| Current Smoker, n (%) | 9,621 (43%) | 2,204 (43%) | 7,417 (43%) | 0.023 | 0.051 |
| Drinker, n (%) | 6,793 (70%) | 1,895 (60%) | 4,898 (68%) | <0.001 | 0.176 |
| Physically Active, n (%) | 8,999 (40%) | 2,678 (50%) | 6,321 (37%) | <0.001 | 0.166 |
| Height (cm) | 168 (160, 175) | 170 (163, 177) | 166 (159, 173) | <0.001 | 0.218 |
| Weight (kg/m²) | 78 (66, 93) | 81 (75, 88) | 77 (65, 92) | <0.001 | 0.041 |
| Waist (cm) | 98 (88, 109) | 97 (86, 108) | 99 (89, 110) | <0.001 | 0.166 |

Note: Data are presented as median (interquartile range) for continuous variables and n (%) for categorical variables. *P* values were derived from the Wilcoxon rank-sum test for continuous variables and the Pearson’s chi-squared test or Fisher's exact test for categorical variables.

Abbreviations: BMI, Body Mass Index.

**Supplementary Table S4. Principal Component Analysis Loadings, Eigenvalues, and Variance Explained for IRD-PS and SLI-PS (First Principal Component)**

| **Pathological** **Axis** | **Variable** | **Shaanxi Dataset** | |  | | **NHANES** | |  | |
| --- | --- | --- | --- | --- | --- | --- | --- | --- | --- |
|  |  | **Loading (PC1)** | **Variance explained (PC1, %)** | | **Eigenvalue** | **Loading (PC1)** | **Variance explained (PC1, %)** | | **Eigenvalue** |
| IRD-PS | TyG | 0.867 | 65.9 | | 1.977 | 0.897 | 60.7 | | 1.821 |
|  | HOMA-IR | - |  | |  | 0.527 |  | |  |
|  | TG/HDL-C | 0.871 |  | |  | 0.860 |  | |  |
|  | HDL-C (negated) | 0.683 |  | |  | - |  | |  |
|  |  |  |  | |  |  |  | |  |
| SLI-PS | WBC | 0.954 | 51.2 | | 2.048 | 0.734 | 71.7 | | 2.151 |
|  | NE | 0.918 |  | |  | - |  | |  |
|  | NLR | - |  | |  | 0.885 |  | |  |
|  | PLT | 0.457 |  | |  | - |  | |  |
|  | PLR | - |  | |  | 0.910 |  | |  |
|  | UA | 0.295 |  | |  | - |  | |  |

Abbreviations: HDL-C, High-Density Lipoprotein Cholesterol; IRD-PS, Insulin Resistance/Dyslipidemia Pathological Score; NE, Neutrophils Count; PLT, Platelet Count; SLI-PS, Systemic Low-Grade Inflammation Pathological Score; TG, Triglycerides; TyG, Triglyceride-Glucose Index; UA, Uric Acid; WBC, White Blood Cell Count; NLR, Neutrophil-to-Lymphocyte Ratio; HOMA-IR, Homeostasis Model Assessment of Insulin Resistance; PLR, Platelet-to-Lymphocyte Ratio; PC1, First Principal Component.

**Supplementary Table S4. Baseline Characteristics of Participants by Metabolic Phenotype (Weighted NHANES Data)**

| **Characteristic** | **Overall**  **(N = 64,941,968)**  **(n = 5,359)** | **MHNO**  **(N = 18,703,925)**  **(n = 1,560)** | **MUNO**  **(N = 1,369,974)**  **(n = 139)** | **MHO**  **(N = 24,040,534)**  **(n = 1,923)** | **MUO**  **(N = 20,827,534)**  **(n = 1,737)** | ***P* value** |
| --- | --- | --- | --- | --- | --- | --- |
| **Sociodemographic Characteristics** | | | | | | |
| Age (years) | 41 (30, 50) | 35 (25, 47) | 52 (44, 56) | 38 (29, 48) | 45 (36, 53) | **<0.001** |
| Sex (Male), n (%) | 34,000,892  (52%) | 8,644,515  (46%) | 660,577  (48%) | 13,364,232  (56%) | 11,331,568  (54%) | **<0.001** |
| Race/Ethnicity, n (%) |  |  |  |  |  | **<0.001** |
| Mexican American | 6,303,494 (9.7%) | 1,113,075 (6.0%) | 66,533 (4.9%) | 2,932,946 (12%) | 2,190,941 (11%) |  |
| Non-Hispanic Asian people | 3,636,642 (5.6%) | 1,827,639 (9.8%) | 270,577 (20%) | 655,807 (2.7%) | 882,619 (4.2%) |  |
| Non-Hispanic Black people | 7,031,091 (11%) | 1,651,017 (8.8%) | 72,606 (5.3%) | 3,095,852 (13%) | 2,211,615 (11%) |  |
| Non-Hispanic White people | 40,876,616  (63%) | 12,076,491  (65%) | 826,685  (60%) | 14,565,525  (61%) | 13,407,916  (64%) |  |
| Other/Multi-Racial | 7,094,124 (11%) | 2,035,704 (11%) | 133,573 (9.8%) | 2,790,404 (12%) | 2,134,444 (10%) |  |
| Education Level, n (%) |  |  |  |  |  | **<0.001** |
| Less than 9th grade | 2,562,099 (3.9%) | 474,422 (2.5%) | 54,103 (3.9%) | 1,127,612 (4.7%) | 905,962 (4.3%) |  |
| 9-11th grade | 6,433,410 (9.9%) | 1,845,567 (9.9%) | 164,360 (12%) | 2,040,384 (8.5%) | 2,383,100 (11%) |  |
| High school/GED | 13,994,295 (22%) | 3,652,945 (20%) | 348,143 (25%) | 5,065,484 (21%) | 4,927,723 (24%) |  |
| Some college/AA degree | 21,084,072 (32%) | 5,465,369 (29%) | 330,800 (24%) | 8,067,764 (34%) | 7,220,139 (35%) |  |
| College graduate or above | 20,868,093 (32%) | 7,265,622 (39%) | 472,569 (34%) | 7,739,291 (32%) | 5,390,611 (26%) |  |
| Marital Status, n (%) |  |  |  |  |  | **<0.001** |
| Married/Living with partner | 40,646,392  (63%) | 10,544,982 (56%) | 936,143  (68%) | 15,479,515  (65%) | 13,685,752  (65%) |  |
| Divorced/Separated/Widowed | 8,720,770  (13%) | 1,941,709  (10%) | 232,022  (17%) | 2,995,848  (12%) | 3,551,192  (17%) |  |
| Never married | 15,574,806  (24%) | 6,217,235  (33%) | 201,809  (15%) | 5,565,171  (23%) | 3,590,591  (17%) |  |
| **Lifestyle Factors** | | | | | | |
| Current Smoker, n (%) | 27,865,429 (43%) | 7,717,022 (41%) | 759,149 (55%) | 9,441,403 (39%) | 9,947,854 (48%) | **<0.001** |
| Drinker, n (%) | 39,145,555  (60%) | 11,210,338 (60%) | 904,369  (66%) | 14,437,121  (60%) | 12,593,728  (60%) | 0.8 |
| Physically Active, n (%) | 32,311,120  (50%) | 8,708,924  (47%) | 736,874  (54%) | 11,868,515  (49%) | 10,996,807  (53%) | **0.044** |
| **Anthropometrics** | | | | | | |
| Height (cm) | 170 (163, 177) | 169 (163, 176) | 169 (163, 177) | 170 (163, 177) | 170 (163, 178) | **0.049** |
| BMI (kg/m²) | 28 (24, 33) | 22 (21, 24) | 23 (22, 24) | 29 (27, 33) | 32 (29, 37) | **<0.001** |
| Waist (cm) | 97 (86, 108) | 81 (76, 86) | 90 (84, 95) | 99 (93, 107) | 109 (102, 119) | **<0.001** |
| **Blood Pressure** | | | | | | |
| SBP (mmHg) | 117 (109, 127) | 112 (105, 119) | 127 (116, 136) | 115 (108, 123) | 125 (116, 135) | **<0.001** |
| DBP (mmHg) | 71 (65, 78) | 67 (62, 74) | 75 (65, 83) | 70 (65, 76) | 77 (69, 83) | **<0.001** |
| **Laboratory Tests** | | | | | | |
| TC (mg/dL) | 188 (164, 215) | 180 (158, 204) | 201 (173, 231) | 186 (164, 213) | 197 (172, 226) | **<0.001** |
| TG (mg/dL) | 95 (63, 145) | 69 (49, 99) | 166 (115, 229) | 87 (62, 118) | 153 (100, 217) | **<0.001** |
| HDL-C (mg/dL) | 51 (42, 61) | 60 (50, 71) | 45 (38, 54) | 52 (45, 60) | 42 (36, 49) | **<0.001** |
| LDL-C (mg/dL) | 111 (90, 136) | 102 (82, 123) | 116 (90, 138) | 114 (93, 137) | 118 (96, 143) | **<0.001** |
| Glucose (mg/dL) | 99 (93, 107) | 94 (89, 99) | 106 (101, 112) | 97 (92, 103) | 105 (100, 114) | **<0.001** |
| Insulin (µU/mL) | 9 (6, 15) | 5 (4, 8) | 8 (6, 11) | 9 (6, 13) | 15 (10, 22) | **<0.001** |
| HbA1c (%) | 5.40 (5.10, 5.60) | 5.20 (5.00, 5.40) | 5.50 (5.30, 5.80) | 5.30 (5.10, 5.50) | 5.60 (5.30, 5.90) | **<0.001** |
| Cr (mg/dL) | 0.83 (0.70, 0.96) | 0.81 (0.69, 0.94) | 0.76 (0.64, 0.93) | 0.84 (0.72, 0.98) | 0.83 (0.71, 0.96) | **<0.001** |
| UA (mg/dL) | 5.30 (4.40, 6.30) | 4.80 (3.90, 5.60) | 5.00 (4.20, 6.10) | 5.40 (4.50, 6.30) | 5.90 (4.90, 6.80) | **<0.001** |
| CK (U/L) | 108 (75, 170) | 98 (69, 152) | 84 (60, 151) | 113 (77, 178) | 113 (78, 177) | **<0.001** |
| WBC (10⁹/L) | 6.40 (5.50, 7.90) | 6.00 (5.10, 7.20) | 6.70 (5.60, 7.80) | 6.40 (5.40, 7.60) | 7.20 (6.00, 8.40) | **<0.001** |
| Neutrophils (%) | 58 (51, 63) | 56 (50, 62) | 58 (52, 65) | 57 (51, 63) | 59 (52, 64) | **<0.001** |
| Lymphocytes (%) | 31 (26, 36) | 32 (27, 37) | 29 (24, 35) | 31 (26, 36) | 30 (25, 35) | **0.001** |
| PLT (10⁹/L) | 231 (199, 270) | 228 (196, 261) | 242 (201, 274) | 231 (198, 270) | 237 (204, 278) | **<0.001** |
| Urine Protein (mg/dL) | 7 (4, 14) | 7 (4, 12) | 6 (3, 13) | 7 (4, 12) | 9 (5, 17) | **<0.001** |
| Urine Creatinine (mg/dL) | 116 (68, 175) | 102 (55, 161) | 76 (49, 149) | 123 (73, 182) | 126 (76, 182) | **<0.001** |
| **Imaging/Body Composition** | | | | | | |
| DXA Visceral Fat Mass (g) | 470 (287, 685) | 241 (175, 324) | 427 (320, 616) | 481 (351, 634) | 721 (549, 907) | **<0.001** |
| DXA Visceral Fat Volume (cm³) | 508 (311, 741) | 260 (190, 350) | 462 (346, 666) | 520 (380, 686) | 779 (594, 981) | **<0.001** |
| DXA Visceral Fat Area (cm²) | 97 (60, 142) | 50 (36, 67) | 89 (66, 128) | 100 (73, 132) | 149 (114, 188) | **<0.001** |
| **Calculated Indices** | | | | | | |
| eGFR (mL/min/1.73m²) | 111 (101, 122) | 115 (105, 126) | 109 (101, 114) | 111 (100, 122) | 109 (99, 118) | **<0.001** |
| HOMA-IR | 2.24 (1.36, 3.98) | 1.29 (0.84, 1.90) | 2.12 (1.64, 3.38) | 2.14 (1.46, 3.28) | 4.15 (2.69, 6.46) | **<0.001** |
| TyG Index | 8.46 (8.02, 8.91) | 8.09 (7.74, 8.45) | 9.05 (8.50, 9.35) | 8.36 (8.00, 8.68) | 8.99 (8.58, 9.41) | **<0.001** |
| NLR | 1.85  (1.42, 2.42) | 1.78  (1.36, 2.30) | 1.99  (1.44, 2.72) | 1.83  (1.42, 2.36) | 1.97  (1.50, 2.57) | **<0.001** |
| PLR | 751  (597, 959) | 710  (565, 916) | 773  (622, 1064) | 757  (589, 950) | 785  (634, 1012) | **<0.001** |
| VA (log-transformed) | 4.59 (4.10, 4.96) | 3.93 (3.62, 4.22) | 4.50 (4.21, 4.86) | 4.61 (4.30, 4.89) | 5.01 (4.74, 5.24) | **<0.001** |
| SLI-PS | -0.17  (-1.21, 1.25) | -0.59  (-1.56, 0.72) | 0.08  (-1.15, 1.66) | -0.22  (-1.26, 1.15) | 0.28  (-0.83, 1.72) | **<0.001** |
| IRD-PS | -0.06  (-0.95, 1.02) | -0.86  (-1.51, -0.14) | 1.27  (0.27, 2.22) | -0.28  (-0.99, 0.42) | 1.26  (0.25, 2.50) | **<0.001** |
| **Outcomes / Groupings** | | | | | | |
| MetS, n (%) | 22,197,508  (34%) | 0  (0%) | 1,369,974 (100%) | 0  (0%) | 20,827,534  (100%) | **<0.001** |
| CKM Stage (3-level), n (%) |  |  |  |  |  | **<0.001** |
| Early (Stage 0/1) | 26,194,055  (40%) | 12,488,566 (67%) | 0  (0%) | 13,705,489 (57%) | 0  (0%) |  |
| Middle (Stage 2) | 35,985,462  (55%) | 5,630,445  (30%) | 1,344,075  (98%) | 9,586,287  (40%) | 19,424,655 (93%) |  |
| Late (Stage 3/4) | 2,762,451  (4.3%) | 584,914  (3.1%) | 25,899  (1.9%) | 748,759  (3.1%) | 1,402,879  (6.7%) |  |

Note: Data are presented as Median (Interquartile Range) for continuous variables and n (%) for categorical variables. In the column headers, n denotes the unweighted sample size (the actual number of participants in the survey), while N denotes the weighted sample size (the estimated number of individuals in the U.S. population represented by the sample). *P* values were calculated using the design-based Kruskal-Wallis test for continuous variables and the Rao & Scott adjusted Pearson’s Chi-square test for categorical variables.
Abbreviations: BMI, body mass index; CK, creatine kinase; CKM, cardiovascular-kidney-metabolic; Cr, creatinine; DBP, diastolic blood pressure; DXA, dual-energy X-ray absorptiometry; eGFR, estimated glomerular filtration rate; HbA1c, glycated hemoglobin; HDL-C, high-density lipoprotein cholesterol; HOMA-IR, Homeostatic Model Assessment for Insulin Resistance; IRD-PS, Insulin Resistance/Dyslipidemia Pathological Score; LDL-C, low-density lipoprotein cholesterol; VA (log-transformed),log-transformed Visceral Adiposity; MetS, Metabolic Syndrome; NE, Neutrophils; NLR, Neutrophil-to-Lymphocyte Ratio; PLT, Platelets; PLR, Platelet-to-Lymphocyte Ratio; SBP, Systolic Blood Pressure; SLI-PS, Systemic Low-grade Inflammation Pathological Score; TC, Total Cholesterol; TG, Triglycerides; TyG Index, Triglyceride-Glucose Index; UA, Uric Acid; VA, Visceral Adipose; WBC, White Blood Cell.

**Supplementary** **Table S5. Study Population Characteristics Stratified by CKM Stage in Shaanxi Dataset**

| **Characteristic** | **Overall**  **(n = 2,100)** | **Early (Stage 0-1)**  **(n = 608)** | **Mid (Stage 2)**  **(n = 646)** | **Late (Stage 3-4)**  **(n = 846)** | ***P* value** | **SMD** |
| --- | --- | --- | --- | --- | --- | --- |
| **Sociodemographic Characteristics** | | | | | | |
| Age (years) | 42 (31, 55) | 32 (23.75, 41) | 40 (32, 48) | 55 (44, 66) | **<0.001** | 0.956 |
| Sex, n (%) |  |  |  |  | **<0.001** | 0.346 |
| Male | 955 (45.5%) | 185 (30.4%) | 358 (55.4%) | 412 (48.7%) |  |  |
| Female | 1145 (54.5%) | 423 (69.6%) | 288 (44.6%) | 434 (51.3%) |  |  |
| Education Level, n (%) |  |  |  |  | **<0.001** | 0.659 |
| Primary or below | 770 (36.7%) | 103 (16.9%) | 177 (27.4%) | 490 (57.9%) |  |  |
| Junior high graduation | 471 (22.4%) | 139 (22.9%) | 167 (25.9%) | 165 (19.5%) |  |  |
| Secondary graduate | 303 (14.4%) | 120 (19.7%) | 106 (16.4%) | 77 (9.1%) |  |  |
| Junior college and above | 556 (26.5%) | 246 (40.5%) | 196 (30.3%) | 114 (13.5%) |  |  |
| **Lifestyle Factors** | | | | | | |
| Lack of activity, n (%) | 346 (16.5) | 113 (18.6) | 96 (14.9) | 137 (16.2) | 0.188 | 0.068 |
| Current Smoker, n (%) | 296 (14.1%) | 41 (6.8%) | 96 (14.9%) | 159 (18.8%) | **<0.001** | 0.245 |
| Drinker, n (%) | 392 (18.7%) | 67 (11.1%) | 138 (21.4%) | 187 (22.1%) | **<0.001** | 0.201 |
| **Anthropometrics** | | | | | | |
| Height (cm) | 162.00  (157.50, 169.00) | 162.00  (158.00, 168.00) | 165.00  (159.00, 170.45) | 161.50  (156.00, 168.00) | **<0.001** | 0.225 |
| BMI (kg/m²) | 22.87 (20.63, 25.30) | 21.74 (19.83, 23.74) | 23.68 (21.68, 26.17) | 23.13 (20.86, 25.47) | **<0.001** | 0.406 |
| Waist (cm) | 80.00 (75.00, 88.00) | 77.00 (72.00, 83.00) | 82.00 (77.00, 89.30) | 82.35 (77.00, 89.00) | **<0.001** | 0.405 |
| **Laboratory Tests** | | | | | | |
| TC (mmol/L) | 4.27 (3.75, 4.86) | 3.93 (3.51, 4.31) | 4.56 (3.94, 5.11) | 4.44 (3.88, 5.04) | **<0.001** | 0.587 |
| TG (mmol/L) | 1.25 (0.85, 1.97) | 0.86 (0.62, 1.12) | 1.82 (1.19, 2.61) | 1.40 (0.95, 2.19) | **<0.001** | 0.817 |
| HDL-C (mmol/L) | 1.37 (1.21, 1.57) | 1.45 (1.32, 1.60) | 1.29 (1.13, 1.48) | 1.37 (1.22, 1.57) | **<0.001** | 0.370 |
| LDL-C (mmol/L) | 2.42 (2.01, 2.92) | 2.20 (1.85, 2.52) | 2.64 (2.19, 3.20) | 2.49 (2.04, 2.98) | **<0.001** | 0.526 |
| Glucose (mmol/L) | 4.94 (4.60, 5.44) | 4.73 (4.47, 5.02) | 4.94 (4.62, 5.45) | 5.12 (4.73, 5.92) | **<0.001** | 0.488 |
| HbA1c (%) | 5.40 (5.20, 5.70) | 5.30 (5.10, 5.50) | 5.50 (5.20, 5.70) | 5.50 (5.20, 5.90) | **<0.001** | 0.360 |
| Cr (μmol/L) | 61.00 (53.00, 73.00) | 58.00 (51.00, 67.00) | 63.00 (55.00, 74.00) | 63.00 (53.00, 75.00) | **<0.001** | 0.241 |
| UA (μmol/L) | 320.00  (266.00, 384.25) | 293.00  (245.00, 355.00) | 339.00  (282.00, 402.00) | 323.50  (272.25, 388.00) | **<0.001** | 0.308 |
| UACR (mg/g) | 3.91 (1.88, 8.51) | 3.26 (1.67, 4.94) | 3.97 (2.00, 8.15) | 4.98 (2.07, 14.53) | **<0.001** | 0.377 |
| WBC (×10⁹/L) | 5.88 (4.95, 6.80) | 5.80 (4.87, 6.70) | 6.00 (4.99, 6.92) | 5.82 (5.01, 6.76) | **0.023** | 0.118 |
| TyG index | 8.52 (8.09, 9.07) | 8.07 (7.75, 8.37) | 8.89 (8.46, 9.29) | 8.67 (8.24, 9.25) | **<0.001** | 0.960 |
| **Instrument-based Examination** | | | | | | |
| SBP (mmHg) | 122 (111, 136) | 113 (105, 121) | 124 (114, 136) | 130 (116, 146) | **<0.001** | 0.796 |
| DBP (mmHg) | 79 (72, 87) | 74 (70, 80) | 82 (74, 89) | 81 (74, 91) | **<0.001** | 0.567 |
| VA Grade | 8 (6, 11) | 7 (5, 10) | 8 (6, 11) | 8 (6, 11) | **<0.001** | 0.245 |
| EF (%) | 67.29 (62.64, 71.43) | 68.35 (63.89, 72.22) | 68.11 (63.90, 71.90) | 65.67 (60.00, 70.31) | **<0.001** | 0.344 |
| baPWV (cm/s) | 1256.25  (1146.00, 1448.25) | 1170.00  (1082.00, 1231.38) | 1260.50  (1163.50, 1394.25) | 1416.00  (1217.62, 1682.25) | **<0.001** | 0.901 |
| ABI | 1.07 (1.02, 1.12) | 1.09 (1.04, 1.15) | 1.07 (1.02, 1.12) | 1.05 (1.00, 1.10) | **<0.001** | 0.295 |
| eGFR (mL/min/1.73m²) | 124.61  (110.03, 139.60) | 138.27  (126.44, 149.45) | 124.22  (114.02, 136.70) | 113.27  (102.44, 128.30) | **<0.001** | 0.781 |
| LVMI (g/m²) | 78.56 (68.01, 92.41) | 71.57 (62.50, 81.93) | 78.38 (67.99, 92.33) | 84.37 (73.37, 100.56) | **<0.001** | 0.264 |
| **Disease History / Medication** | | | | | | |
| Hypertension, n (%) | 608 (29.0%) | 0 (0.0%) | 224 (34.7%) | 384 (45.4%) | **<0.001** | 0.847 |
| Diabetes, n (%) | 204 (9.7%) | 0 (0.0%) | 70 (10.8%) | 134 (15.8%) | **<0.001** | 0.418 |
| Dyslipidemia, n (%) | 955 (47.8%) | 0 (0.0%) | 504 (79.6%) | 451 (54.9%) | **<0.001** | 1.634 |
| Proteinuria, n (%) | 171 (8.1%) | 0 (0.0%) | 45 (7.0%) | 126 (14.9%) | **<0.001** | 0.412 |
| Antihypertensive Drug, n (%) | 161 (7.7%) | 0 (0.0%) | 36 (5.6%) | 125 (14.8%) | **<0.001** | 0.413 |
| Antidiabetic Drug, n (%) | 36 (1.7%) | 0 (0.0%) | 10 (1.5%) | 26 (3.1%) | **<0.001** | 0.177 |
| Dyslipidemia Drug, n (%) | 33 (1.6%) | 0 (0.0%) | 10 (1.5%) | 23 (2.7%) | **<0.001** | 0.165 |
| **Core Indices** | | | | | | |
| IRD-PS | -0.18 (-0.94, 0.72) | -0.86 (-1.33, -0.32) | 0.49 (-0.29, 1.23) | -0.02 (-0.72, 0.98) | **<0.001** | 0.801 |
| SLI-PS | -0.16 (-1.02, 0.79) | -0.23 (-1.10, 0.71) | 0.01 (-0.96, 0.94) | -0.20 (-1.01, 0.73) | **0.023** | 0.107 |
| **Groups** | | | | | | |
| MHO Phenotype, n (%) |  |  |  |  | **<0.001** | 0.711 |
| MHNO | 1149 (54.7%) | 472 (77.6%) | 280 (43.3%) | 397 (46.9%) |  |  |
| MHO | 522 (24.9%) | 136 (22.4%) | 199 (30.8%) | 187 (22.1%) |  |  |
| MUNO | 179 (8.5%) | 0 (0.0%) | 71 (11.0%) | 108 (12.8%) |  |  |
| MUO | 250 (11.9%) | 0 (0.0%) | 96 (14.9%) | 154 (18.2%) |  |  |

Note: Data are presented as median (interquartile range) for continuous variables and n (%) for categorical variables. P values were derived from the Kruskal-Wallis test for continuous variables and the chi-square test or Fisher’s exact test for categorical variables. SMD, Standardized Mean Difference; an SMD ≥ 0.2 indicates a meaningful imbalance.

Abbreviation: ABI: Ankle-Brachial Index; baPWV: Brachial-Ankle Pulse Wave Velocity; BMI: Body Mass Index; CKM: Cardiovascular-Kidney-Metabolic Syndrome; Cr: Creatinine; DBP: Diastolic Blood Pressure; EF: Ejection Fraction; eGFR: estimated Glomerular Filtration Rate; HbA1c: Glycated Hemoglobin A1c; HDL-C: High-Density Lipoprotein Cholesterol; IRD-PS: Insulin Resistance/Dyslipidemia Pathological Score; LDL-C: Low-Density Lipoprotein Cholesterol; LVMI: Left Ventricular Mass Index; MHO: Metabolically Healthy Obese; MHNO: Metabolically Healthy Non-Obese; MUO: Metabolically Unhealthy Obese; MUNO: Metabolically Unhealthy Non-Obese; SBP: Systolic Blood Pressure; SLI-PS: Systemic Low-grade Inflammation Pathological Score; TC: Total Cholesterol; TG: Triglycerides; UA: Uric Acid; UACR: Urinary Albumin-to-Creatinine Ratio; VA: Visceral Adiposity; WBC: White Blood Cell Count.

**Supplementary Table S6. Baseline Characteristics of Participants by CKM Stage (Weighted NHANES Data)**

| **Characteristic** | **Early Stage**  **(N = 26,194,055)**  **(n = 2,068)** | **Middle Stage**  **(N = 35,985,463)**  **(n = 3,032)** | **Late Stage**  **(N = 2,762,451)**  **(n = 259)** | **Overall**  **(N = 64,941,968)**  **(n = 5,393)** | ***P* value** |
| --- | --- | --- | --- | --- | --- |
| **Sociodemographic Characteristics** | | | | | |
| Age (years) | 34 (26, 44) | 44 (34, 53) | 51 (45, 56) | 41 (30, 50) | **<0.001** |
| Sex (Female), n (%) | 14,074,722 (54) | 15,601,688 (43) | 1,264,666 (46) | 30,941,076 (48) | **<0.001** |
| Race/Ethnicity, n (%) |  |  |  |  | **0.007** |
| Mexican American | 2,472,866 (9.4) | 3,657,087 (10) | 173,542 (6.3) | 6,303,494 (9.7) |  |
| Non-Hispanic Asian people | 1,637,750 (6.3) | 1,940,861 (5.4) | 58,031 (2.1) | 3,636,642 (5.6) |  |
| Non-Hispanic Black people | 2,485,758 (9.5) | 4,071,284 (11) | 474,049 (17) | 7,031,091 (11) |  |
| Non-Hispanic White people | 16,657,383 (64) | 22,541,474 (63) | 1,677,760 (61) | 40,876,616 (63) |  |
| Other/Multi-Racial | 2,940,298 (11) | 3,774,756 (10) | 379,069 (14) | 7,094,124 (11) |  |
| Education Level, n (%) |  |  |  |  | **<0.001** |
| Less than 9th grade | 924,570 (3.5) | 1,501,039 (4.2) | 136,490 (4.9) | 2,562,099 (3.9) |  |
| 9-11th grade | 1,906,154 (7.3) | 4,066,289 (11) | 460,967 (17) | 6,433,410 (9.9) |  |
| High school/GED | 5,112,356 (20) | 8,001,791 (22) | 880,148 (32) | 13,994,295 (22) |  |
| Some college/AA degree | 7,723,888 (29) | 12,530,507 (35) | 829,677 (30) | 21,084,072 (32) |  |
| College graduate or above | 10,527,087 (40) | 9,885,836 (27) | 455,170 (16) | 20,868,093 (32) |  |
| Marital Status, n (%) |  |  |  |  | **<0.001** |
| Divorced | 1,832,301 (7.0) | 3,925,022 (11) | 530,005 (19) | 6,287,329 (9.7) |  |
| Living with partner | 3,193,768 (12) | 3,414,774 (9.5) | 291,397 (11) | 6,899,938 (11) |  |
| Married | 12,774,228 (49) | 19,549,033 (54) | 1,423,193 (52) | 33,746,454 (52) |  |
| Never married | 7,776,976 (30) | 7,419,523 (21) | 378,307 (14) | 15,574,806 (24) |  |
| Separated/Widowed | 616,782 (2.4) | 1,677,110 (4.7) | 139,549 (5.1) | 2,433,441 (3.7) |  |
| **Lifestyle Factors** | | | | | |
| Current Smoker, n (%) | 9,414,832 (36) | 16,540,576 (46) | 1,910,021 (69) | 27,865,429 (43) | **<0.001** |
| Drinker, n (%) | 15,620,813 (60) | 21,660,040 (60) | 1,864,703 (68) | 39,145,555 (60) | 0.3 |
| Physically Active, n (%) | 12,441,217 (47) | 18,512,131 (51) | 1,357,771 (49) | 32,311,120 (50) | 0.12 |
| **Anthropometrics** | | | | | |
| Height (cm) | 169 (163, 176) | 171 (163, 178) | 169 (162, 177) | 170 (163, 177) | **0.006** |
| Weight (kg) | 73 (63, 86) | 87 (74, 102) | 86 (71, 104) | 81 (68, 96) | **<0.001** |
| BMI (kg/m²) | 25 (22, 29) | 30 (26, 34) | 29 (26, 36) | 28 (24, 33) | **<0.001** |
| Waist (cm) | 89 (81, 98) | 102 (93, 113) | 103 (92, 119) | 97 (86, 108) | **<0.001** |
| **Blood Pressure** |  |  |  |  |  |
| SBP (mmHg) | 111 (105, 117) | 123 (114, 132) | 123 (115, 135) | 117 (109, 127) | **<0.001** |
| DBP (mmHg) | 67 (62, 72) | 75 (68, 82) | 75 (67, 83) | 71 (65, 78) | **<0.001** |
| **Laboratory Tests** | | | | | |
| TC (mg/dL) | 178 (156, 203) | 195 (171, 223) | 187 (163, 221) | 188 (164, 215) | **<0.001** |
| TG (mg/dL) | 71 (52, 94) | 130 (83, 185) | 112 (75, 167) | 95 (63, 145) | **<0.001** |
| HDL-C (mg/dL) | 57 (48, 66) | 47 (39, 57) | 48 (40, 58) | 51 (42, 61) | **<0.001** |
| LDL-C (mg/dL) | 105 (85, 127) | 116 (94, 141) | 110 (86, 133) | 111 (90, 136) | **<0.001** |
| Glucose (mg/dL) | 95 (90, 101) | 102 (95, 110) | 105 (95, 117) | 99 (93, 107) | **<0.001** |
| Insulin (µU/mL) | 7 (5, 10) | 12 (7, 18) | 11 (6, 20) | 9 (6, 15) | **<0.001** |
| HbA1c (%) | 5.30 (5.00, 5.40) | 5.50 (5.20, 5.80) | 5.70 (5.40, 6.30) | 5.40 (5.10, 5.60) | **<0.001** |
| Cr (mg/dL) | 0.82 (0.70, 0.94) | 0.83 (0.71, 0.97) | 0.85 (0.71, 0.99) | 0.83 (0.70, 0.96) | 0.2 |
| UA (mg/dL) | 5.00 (4.20, 5.80) | 5.70 (4.70, 6.50) | 5.70 (4.70, 6.40) | 5.30 (4.40, 6.30) | **<0.001** |
| CK (U/L) | 102 (72, 166) | 111 (78, 173) | 109 (72, 155) | 108 (75, 170) | **0.004** |
| WBC (10⁹/L) | 6.10 (5.20, 7.40) | 6.70 (5.70, 8.20) | 7.10 (6.00, 8.20) | 6.40 (5.50, 7.90) | **<0.001** |
| Neutrophils (%) | 56 (51, 62) | 58 (52, 64) | 60 (54, 65) | 58 (51, 63) | **<0.001** |
| Lymphocytes (%) | 32 (27, 37) | 30 (25, 36) | 28 (23, 33) | 31 (26, 36) | **<0.001** |
| PLT (10⁹/L) | 228 (197, 263) | 235 (201, 275) | 223 (203, 270) | 231 (199, 270) | **<0.001** |
| Urine Protein (mg/dL) | 6 (4, 11) | 8 (4, 16) | 10 (5, 31) | 7 (4, 14) | **<0.001** |
| Urine Creatinine (mg/dL) | 116 (65, 175) | 118 (70, 176) | 105 (59, 164) | 116 (68, 175) | 0.3 |
| **Imaging / Body Composition** | | | | | |
| DXA Visceral Fat Mass (g) | 319 (219, 470) | 576 (403, 786) | 648 (463, 895) | 470 (287, 685) | **<0.001** |
| DXA Visceral Fat Volume (cm³) | 345 (237, 508) | 623 (436, 850) | 701 (501, 968) | 508 (311, 741) | **<0.001** |
| DXA Visceral Fat Area (cm²) | 66 (45, 97) | 119 (84, 163) | 134 (96, 186) | 97 (60, 142) | **<0.001** |
| **Calculated Indices** | | | | | |
| eGFR (mL/min/1.73m²) | 116 (104, 126) | 109 (99, 119) | 104 (93, 113) | 111 (101, 122) | **<0.001** |
| HOMA-IR | 1.62 (1.06, 2.48) | 2.99 (1.75, 5.05) | 2.92 (1.47, 5.82) | 2.24 (1.36, 3.98) | **<0.001** |
| TyG Index | 8.11 (7.79, 8.42) | 8.81 (8.32, 9.19) | 8.73 (8.22, 9.16) | 8.46 (8.02, 8.91) | **<0.001** |
| NLR | 1.77 (1.38, 2.25) | 1.91 (1.45, 2.53) | 2.20 (1.66, 2.83) | 1.85 (1.42, 2.42) | **<0.001** |
| PLR | 711 (575, 910) | 777 (617, 997) | 836 (661, 1,068) | 751 (597, 959) | **<0.001** |
| VA (log-transformed) | 4.21 (3.84, 4.59) | 4.79 (4.44, 5.10) | 4.91 (4.58, 5.23) | 4.59 (4.10, 4.96) | **<0.001** |
| IRD-PS | -0.78 (-1.37, -0.18) | 0.75 (-0.35, 1.75) | 0.45 (-0.55, 1.80) | -0.06 (-0.95, 1.02) | **<0.001** |
| SLI-PS | -0.55 (-1.51, 0.72) | 0.10 (-1.00, 1.55) | 0.63 (-0.51, 2.05) | -0.17 (-1.21, 1.25) | **<0.001** |
| **Phenotype** | | | | | |
| MetS, n (%) | 0 (0) | 20,768,730 (58) | 1,428,778 (52) | 22,197,508 (34) | **<0.001** |
| Metabolic Phenotype, n (%) |  |  |  |  | **<0.001** |
| MUNO | 0 (0) | 1,344,075 (3.7) | 25,899 (0.9) | 1,369,974 (2.1) |  |
| MUO | 0 (0) | 19,424,655 (54) | 1,402,879 (51) | 20,827,534 (32) |  |
| MHNO | 12,488,566 (48) | 5,630,445 (16) | 584,914 (21) | 18,703,925 (29) |  |
| MHO | 13,705,489 (52) | 9,586,287 (27) | 748,759 (27) | 24,040,535 (37) |  |

Note: Data are presented as Median (Interquartile Range) for continuous variables and n (%) for categorical variables. In the column headers, n denotes the unweighted sample size (the actual number of participants in the survey), while N denotes the weighted sample size (the estimated number of individuals in the U.S. population represented by the sample).
P values were calculated using the design-based Kruskal-Wallis test for continuous variables and the Rao & Scott adjusted Pearson’s Chi-square test for categorical variables.
Abbreviations: BMI, Body Mass Index; CK, Creatine Kinase; CKM, Cardiovascular-Kidney-Metabolic Syndrome; Cr, Creatinine; DBP, Diastolic Blood Pressure; DXA, Dual-energy X-ray Absorptiometry; eGFR, estimated Glomerular Filtration Rate; HbA1c, Glycated Hemoglobin; HDL-C, High-Density Lipoprotein Cholesterol; HOMA-IR, Homeostatic Model Assessment for Insulin Resistance; IRD-PS, Insulin Resistance/Dyslipidemia Pathological Score; LDL-C, Low-Density Lipoprotein Cholesterol; VA (log-transformed), Log-transformed Visceral Adiposity; MetS, Metabolic Syndrome; NE, Neutrophils; NLR, Neutrophil-to-Lymphocyte Ratio; PLT, Platelets; PLR, Platelet-to-Lymphocyte Ratio; SBP, Systolic Blood Pressure; SLI-PS, Systemic Low-grade Inflammation Pathological Score; TC, Total Cholesterol; TG, Triglycerides; TyG Index, Triglyceride-Glucose Index; UA, Uric Acid; VAT, Visceral Adipose Tissue; WBC, White Blood Cell.

**Supplementary Table S7. IPW-weighted Models for Pathological Axes and CKM Stages in Shaanxi Sample**

| **Variables** | **Established CKM Status** | | **Advanced CKM Status** | |
| --- | --- | --- | --- | --- |
|  | **Weighted OR (95% CI)** | ***P* value** | **Weighted OR (95% CI)** | ***P* value** |
| VA | 1.05 (1.01, 1.09) | **0.006** | 1.03 (1.00, 1.06) | **0.040** |
| IRD-PS | 2.49 (2.18, 2.86) | **<0.001** | 1.02 (0.95, 1.10) | 0.600 |
| SLI -PS | 1.08 (0.99, 1.18) | 0.073 | 1.11 (1.03, 1.19) | **0.009** |

Note: This was a fully adjusted model including age, sex, smoking status, drinking status, physical activity, education level, family history of cardiovascular disease, and family history of stroke. Firth penalization was applied to address data separation. To account for selection bias, Inverse Probability Weighting (IPW) was employed. Weights were calculated as the inverse of the propensity scores obtained from a logistic regression model, which predicted the probability of inclusion based on demographic and lifestyle factors, thereby aligning the analysis sample (n=2,100) with the source population (n=9,336).

**Supplementary Table S8. Population Representativeness and Balance Check via Inverse Probability Weighting (IPW) in Shaanxi Sample**

| **Characteristic** | **Overall Population (n=9,336)** | **Included (IPW Weighted, n=2,100)** | ***P* value** | **SMD** |
| --- | --- | --- | --- | --- |
| **Age (years)** | 43 (31, 58) | 44 (30, 63) | 0.105 | 0.064 |
| **Sex (Male), n (%)** | 4,189 (45%) | 922 (43.9%) | 0.502 | 0.030 |
| **Education Level, n (%)** |  |  | 0.055 |  |
| Primary or below | 3,265 (35%) | 695 (33.1%) |  | 0.037 |
| Junior high graduation | 2,326 (25%) | 527 (25.1%) |  | 0.004 |
| Secondary graduate | 1,474 (16%) | 330 (15.7%) |  | 0.001 |
| Junior college and above | 2,271 (24%) | 544 (25.9%) |  | 0.037 |
| **Lack of activity, n (%)** | 1,093 (12%) | 271 (12.9%) | 0.263 | 0.034 |
| **Current Smoker, n (%)** | 1,624 (18%) | 359 (17.1%) | 0.508 | 0.013 |
| **Drinker, n (%)** | 1,542 (17%) | 334 (15.9%) | 0.763 | 0.023 |
| **Height (cm)** | 162 (156, 169) | 162 (156, 169) | 0.558 | 0.013 |
| **BMI (kg/m^2^)** | 23.9 (21.5, 26.5) | 23.9 (21.4, 26.4) | 0.694 | 0.014 |
| **Waist (cm)** | 85 (77, 94) | 85 (78, 94) | 0.458 | 0.022 |

Note: Continuous variables are expressed as median (IQR), and categorical variables as n (%). For the IPW-weighted sample, n represents the weighted pseudo-count (n*_weighted_* = total n * weighted proportion). *P* values were derived from the weighted Wilcoxon test or weighted Chi-squared test. SMD (Standardized Mean Difference) < 0.1 indicates a balanced distribution between the weighted analysis sample and the source population.

**Supplementary Table S9. Raw counts of participants by Quartiles of Pathological Axes and CKM Stage in Shaanxi Sample**

| **Characteristic** | **Overall** | **Reference Group** | **Case Group** |
| --- | --- | --- | --- |
| **Established CKM Status** | | | |
| VA Quartiles |  |  |  |
| Q1 (Lowest) | 751 (36%) | 279 (46%) | 472 (32%) |
| Q2 | 453 (22%) | 125 (21%) | 328 (22%) |
| Q3 | 504 (24%) | 123 (20%) | 381 (26%) |
| Q4 (Highest) | 389 (19%) | 79 (13%) | 310 (21%) |
| IRD-PS Quartiles |  |  |  |
| Q1 (Lowest) | 525 (25%) | 277 (46%) | 248 (17%) |
| Q2 | 524 (25%) | 214 (35%) | 310 (21%) |
| Q3 | 524 (25%) | 113 (19%) | 411 (28%) |
| Q4 (Highest) | 524 (25%) | 2 (0.3%) | 522 (35%) |
| SLI-PS Quartiles |  |  |  |
| Q1 (Lowest) | 525 (25%) | 167 (28%) | 358 (24%) |
| Q2 | 524 (25%) | 148 (24%) | 376 (25%) |
| Q3 | 524 (25%) | 150 (25%) | 374 (25%) |
| Q4 (Highest) | 524 (25%) | 141 (23%) | 383 (26%) |
| **Advanced CKM Status** | | | |
| VA Quartiles |  |  |  |
| Q1 (Lowest) | 751 (36%) | 465 (37%) | 286 (34%) |
| Q2 | 453 (22%) | 275 (22%) | 178 (21%) |
| Q3 | 504 (24%) | 289 (23%) | 215 (25%) |
| Q4 (Highest) | 389 (19%) | 223 (18%) | 166 (20%) |
| IRD-PS Quartiles |  |  |  |
| Q1 (Lowest) | 525 (25%) | 362 (29%) | 163 (19%) |
| Q2 | 524 (25%) | 309 (25%) | 215 (25%) |
| Q3 | 524 (25%) | 314 (25%) | 210 (25%) |
| Q4 (Highest) | 524 (25%) | 267 (21%) | 257 (30%) |
| SLI-PS Quartiles |  |  |  |
| Q1 (Lowest) | 525 (25%) | 320 (26%) | 205 (24%) |
| Q2 | 524 (25%) | 293 (23%) | 231 (27%) |
| Q3 | 524 (25%) | 315 (25%) | 209 (25%) |
| Q4 (Highest) | 524 (25%) | 324 (26%) | 200 (24%) |

Note: Data are presented as n (%). For Established CKM Status analysis, ‘Reference Group’ corresponds to Early (Stage 0-1) and ‘Case Group’ corresponds to Mid/Late (Stage 2-4). For Advanced CKM Status analysis, ‘Reference Group’ corresponds to Early/Mid (Stage 0-2) and ‘Case Group’ corresponds to Late (Stage 3-4).

Abbreviations: CKM, Cardiovascular-Kidney-Metabolic; IRD-PS, Insulin Resistance/Dyslipidemia Pathological Score; SLI-PS, Systemic Low-Grade Inflammation Pathological Score; VA, Visceral Adiposity.

**Supplementary Table S10. Exploratory Decomposition Analysis of the Association Between Visceral Adiposity and Stage-Specific CKM Risk in Shaanxi Sample**

| **Decomposition Pathway** | **Attenuation Estimate (95% CI)** | ***P* value** | **Prop. Attenuated (95% CI)** | ***P* value** |
| --- | --- | --- | --- | --- |
| **Established CKM Status** | | | | |
| **VA → IRD-PS → Outcome** | **0.0154 (0.0127, 0.0199)** | **<0.001** | **67.1% (52.8%, 87.0%)** | **<0.001** |
| VA → SLI-PS → Outcome | 0.0008 (-0.0001, 0.0020) | 0.086 | 10.0% (-1.0%, 32.0%) | 0.088 |
| Direct Estimate | 0.0076 (0.0024, 0.0129) | **0.004** |  |  |
| Total Estimate | 0.0230 (0.0172, 0.0298) | **<0.001** |  |  |
| **Advanced CKM Status** | | | | |
| VA → IRD-PS → Outcome | 0.0004 (-0.0010, 0.0019) | 0.59 | 7.5% (-28.4%, 53.0%) | 0.596 |
| **VA → SLI-PS → Outcome** | **0.0012 (0.0003, 0.0023)** | **0.004** | **17.9% (3.3%, 64.0%)** | **0.018** |
| Direct Estimate | 0.0054 (0.0005, 0.0104) | **0.036** |  |  |
| Total Estimate | 0.0065 (0.0017, 0.0116) | **0.014** |  |  |

Note: Analyses are based on the fully adjusted model, including age, sex, smoking status, drinking status, physical activity, education level, family history of cardiovascular disease and family history of stroke. Prop. Attenuated is calculated as Attenuation Estimate / Total Estimate.

Abbreviations: CI, Confidence Interval; CKM, Cardiovascular-Kidney-Metabolic; IRD-PS, Insulin Resistance/Dyslipidemia Pathological Score; SLI-PS, Systemic Low-Grade Inflammation Pathological Score; VA, Visceral Adiposity.

**Supplementary Table S11. Exploratory Decomposition Analysis of the Association Between Visceral Adiposity and Stage-Specific CKM Risk in the NHANES**

| **Decomposition Pathway** | **Model 1** | | | **Model 2** | | | **Model 3** | | |
| --- | --- | --- | --- | --- | --- | --- | --- | --- | --- |
|  | **Attenuation**  **Estimate**  **(95% CI)** | ***P* value** | **Prop. Attenuated** | **Attenuation**  **Estimate**  **(95% CI)** | ***P* value** | **Prop. Attenuated** | **Attenuation**  **Estimate**  **(95% CI)** | ***P* value** | **Prop. Attenuated** |
| **Established CKM Status** | | | | | | | | | |
| VA → IRD-PS → Outcome | 0.0011  (0.0005, 0.0020) | **<0.001** | 54.98% | 0.0041  (0.0016, 0.0083) | **<0.001** | 63.9% | 0.0045  (0.0017, 0.0092) | **<0.001** | 64.49% |
| VA → SLI-PS → Outcome | 0.0019  (0.0004, 0.0036) | **0.004** | 3.78% | 0.0051  (0.0018, 0.0090) | **0.008** | 7.33% | 0.0047  (0.0015, 0.0084) | **0.004** | 6.76% |
| Direct Estimate | 0.0009  (0.0004, 0.0015) | **<0.001** |  | 0.0022  (0.0011, 0.0036) | **<0.001** |  | 0.0023  (0.0011, 0.0041) | **<0.001** |  |
| Total Estimate | 0.0019  (0.0009, 0.0035) | **<0.001** |  | 0.0062  (0.0026, 0.0117) | **<0.001** |  | 0.0069  (0.0028, 0.0131) | **<0.001** |  |
| **Advanced CKM Status** | | | | | | | | | |
| VA → IRD-PS → Outcome | 0.0001  (-0.0001, 0.0006) | 0.120 | 9.28% | 0.0015  (-0.0001, 0.0068) | 0.064 | 24.95% | 0.0013  (-0.0001, 0.0063) | 0.056 | 21.20% |
| VA → SLI-PS → Outcome | 0.0001  (0.0001, 0.0005) | **<0.001** | 7.54% | 0.0014  (0.0001, 0.0061) | **0.004** | 20.84% | 0.0009  (0.0001, 0.0040) | **0.032** | 12.92% |
| Direct Estimate | 0.0008  (0.0001, 0.0022) | **<0.001** |  | 0.0004  (-0.0154, 0.0034) | 0.308 |  | 0.0009  (-0.0137, 0.0036) | 0.276 |  |
| Total Estimate | 0.0010  (0.0001, 0.0028) | **<0.001** |  | 0.0020  (-0.0111, 0.0043) | 0.140 |  | 0.0022  (-0.0064, 0.0044) | 0.112 |  |

Note: Results are from a statistical exploratory decomposition analysis. All estimates, 95% CIs, and *P* values were generated using non-parametric bootstrapping (500 simulations).

Abbreviations: CI, Confidence Interval; CKM, Cardiovascular-Kidney-Metabolic; IRD-PS, Insulin Resistance/Dyslipidemia Pathological Score; SLI-PS, Systemic Low-grade Inflammation Pathological Score; VA, Visceral Adiposity.

Model 1: Crude model.
Model 2: Adjusted for age and sex.
Model 3: Fully adjusted model includes age, sex, smoking status, drinking status, physical activity, education level and race/ethnicity.

**Supplementary Table S12. Subgroup Analysis for the Association of Pathological Axes With CKM Stage**

**in Shaanxi Sample**

| **Variable** | **Subgroup** | **No. of Participants** | **Established CKM Status** | | **Advanced CKM Status** | |
| --- | --- | --- | --- | --- | --- | --- |
|  |  |  | **OR (95% CI)** | ***P* value** | **OR (95% CI)** | ***P* value** |
| **VA** | **Overall** | **2100** | **1.14 (1.10, 1.18)** | **<0.001** | **1.04 (1.01, 1.07)** | **0.005** |
|  | **Sex** |  | ***P for interaction = 0.006*** | | ***P for interaction = 0.002*** | |
|  | Male | 955 | 1.07 (1.00, 1.13) | **0.034** | 1.07 (1.02, 1.12) | **0.006** |
|  | Female | 1145 | 1.04 (0.99, 1.09) | 0.090 | 1.00 (0.96, 1.04) | 0.932 |
|  | **Age (Quartiles)** |  | ***P for interaction <0.001*** | | ***P for interaction <0.001*** | |
|  | Q1 | 546 | 1.00 (0.95, 1.06) | 0.891 | 0.97 (0.92, 1.03) | 0.376 |
|  | Q2 | 516 | 1.11 (1.04, 1.20) | **0.003** | 1.08 (1.00, 1.16) | **0.042** |
|  | Q3 | 556 | 1.10 (1.01, 1.19) | **0.028** | 1.07 (1.02, 1.13) | **0.012** |
|  | Q4 | 482 | 1.15 (0.96, 1.39) | 0.129 | 1.10 (1.02, 1.20) | **0.016** |
| **IRD-PS** | **Overall** | **2100** | **2.68 (2.35, 3.06)** | **<0.001** | 1.07 (0.99, 1.15) | 0.078 |
|  | **Sex** |  | ***P for interaction = 0.022*** | | ***P for interaction = 0.008*** | |
|  | Male | 955 | 2.32 (1.90, 2.87) | **<0.001** | 0.96 (0.87, 1.06) | 0.413 |
|  | Female | 1145 | 2.53 (2.11, 3.05) | **<0.001** | 1.12 (0.99, 1.28) | 0.074 |
|  | **Age (Quartiles)** |  | ***P for interaction <0.001*** | | ***P for interaction <0.001*** | |
|  | Q1 | 546 | 2.56 (2.03, 3.29) | **<0.001** | 0.97 (0.76, 1.22) | 0.770 |
|  | Q2 | 516 | 2.91 (2.23, 3.88) | **<0.001** | 1.05 (0.86, 1.25) | 0.640 |
|  | Q3 | 556 | 2.44 (1.89, 3.22) | **<0.001** | 1.16 (1.02, 1.33) | **0.026** |
|  | Q4 | 482 | 1.50 (0.97, 2.47) | 0.069 | 0.90 (0.77, 1.08) | 0.246 |
| **SLI-PS** | **Overall** | **2100** | **1.23 (1.14, 1.33)** | **<0.001** | **1.13 (1.05, 1.21)** | **0.001** |
|  | **Sex** |  | ***P for interaction = 0.021*** | | ***P for interaction = 0.019*** | |
|  | Male | 955 | 1.09 (0.94, 1.26) | 0.249 | 1.09 (0.97, 1.21) | 0.149 |
|  | Female | 1145 | 1.10 (0.98, 1.23) | 0.102 | 1.13 (1.02, 1.25) | **0.022** |
|  | **Age (Quartiles)** |  | ***P for interaction = <0.001*** | | ***P for interaction = <0.001*** | |
|  | Q1 (<31 years) | 546 | 0.98 (0.85, 1.11) | 0.711 | 0.96 (0.82, 1.11) | 0.558 |
|  | Q2 (31~42) | 516 | 1.26 (1.06, 1.51) | **0.010** | 0.97 (0.81, 1.15) | 0.696 |
|  | Q3 (42~55) | 556 | 0.99 (0.82, 1.21) | 0.935 | 1.09 (0.95, 1.26) | 0.211 |
|  | Q4 (>55 years) | 482 | 1.31 (0.89, 2.02) | 0.175 | 1.40 (1.14, 1.75) | **0.001** |

Note: All regression models were estimated using Firth logistic regression. All models were adjusted for age (except age-stratified models), sex (except sex-stratified models), education, smoking, drinking status, physical activity, education level, family history of cardiovascular disease and family history of stroke. Age quartiles are defined as follows: Q1 (Lowest) represents the youngest 25% of the population, Q2 the 25–50% range, Q3 the 50–75% range, and Q4 (Highest) the oldest 25%. Established CKM Status: Mid (Stage 2)/Late (Stage 3-4) vs. Early (Stage 0-1). Advanced CKM Status: Late (Stage 3-4) vs. Early (Stage 0-1)/Mid (Stage 2).
Abbreviations: CI, Confidence Interval; CKM, Cardiovascular-Kidney-Metabolic; IRD-PS, Insulin Resistance/Dyslipidemia Pathological Score; OR, Odds Ratio; SLI-PS, Systemic Low-Grade Inflammation Pathological Score; VA, Visceral Adiposity.

**Supplementary Table S13. Subgroup Analysis for the Association of Pathological Axes With CKM Stage in the NHANES**

| **Variable** | **Subgroup** | **No. of Participants** | **Established CKM Status** | | **Advanced CKM Status** | | |
| --- | --- | --- | --- | --- | --- | --- | --- |
|  |  |  | **OR (95% CI)** | ***P* value** | **OR (95% CI)** | ***P* value** | |
| **IRD-PS** | **Overall** | **5359** | 2.90 (2.60, 3.24) | **<0.001** | 1.10 (1.03, 1.18) | **0.008** | |
|  | **Age (Quartiles)** |  | *P for interaction = 0.096* | | *P for interaction= 0.785* | | |
|  | Q1 (<30 years old) | 1412 | 3.13 (2.61, 3.75) | **<0.001** | 1.25 (1.04, 1.51) | **0.019** | |
|  | Q2 (30~40 years old) | 1339 | 2.87 (2.35, 3.49) | **<0.001** | 1.05 (0.84, 1.31) | 0.655 | |
|  | Q3 (40~50 years old) | 1369 | 3.61 (2.85, 4.57) | **<0.001** | 1.12 (1.02, 1.24) | **0.018** | |
|  | Q4 (>50 years old) | 1239 | 2.39 (1.90, 3.01) | **<0.001** | 1.06 (0.92, 1.23) | 0.424 | |
|  | **Sex** |  | *P for interaction = 0.246* | | *P for interaction = 0.358* | | |
|  | Male | 2719 | 3.03 (2.63, 3.49) | **<0.001** | 1.07 (0.97, 1.19) | 0.162 | |
|  | Female | 2640 | 2.77 (2.39, 3.21) | **<0.001** | 1.13 (1.02, 1.24) | | **0.017** |
|  | **Race/Ethnicity** |  | ***P for interaction < 0.001*** | | *P for interaction = 0.232* | | |
|  | Mexican American | 755 | 3.41 (2.79, 4.18) | **<0.001** | 0.89 (0.68, 1.17) | | 0.383 |
|  | Non-Hispanic Asian people | 760 | 3.96 (3.14, 5.00) | **<0.001** | 1.19 (0.88, 1.62) | | 0.243 |
|  | Non-Hispanic Black people | 1130 | 2.03 (1.73, 2.39) | **<0.001** | 1.15 (1.03, 1.28) | | **0.018** |
|  | Non-Hispanic White people | 1926 | 2.86 (2.44, 3.36) | **<0.001** | 1.10 (0.94, 1.28) | | 0.225 |
|  | Other Hispanic or Multi-Racial | 788 | 3.68 (2.87, 4.71) | **<0.001** | 1.12 (1.04, 1.20) | | **0.003** |
| **SLI-PS** | **Overall** | **5359** | 1.19 (1.13, 1.25) | **<0.001** | 1.09 (1.02, 1.15) | | **0.010** |
|  | **Age (Quartiles)** |  | *P for interaction = 0.348* | | *P for interaction = 0.867* | | |
|  | Q1 (<30 years old) | 1412 | 1.19 (1.11, 1.28) | **<0.001** | 1.06 (0.83, 1.35) | | 0.657 |
|  | Q2 (30~40 years old) | 1339 | 1.14 (1.05, 1.24) | **0.004** | 1.10 (0.98, 1.24) | | 0.088 |
|  | Q3 (40~50 years old) | 1369 | 1.21 (1.10, 1.33) | **<0.001** | 1.05 (0.93, 1.19) | | 0.434 |
|  | Q4 (>50 years old) | 1239 | 1.23 (1.06, 1.42) | **0.007** | 1.10 (1.01, 1.20) | | **0.037** |
|  | **Sex** |  | *P for interaction = 0.102* | | *P for interaction = 0.522* | | |
|  | Male | 2719 | 1.14 (1.07, 1.21) | **<0.001** | 1.07 (0.97, 1.17) | | 0.184 |
|  | Female | 2640 | 1.23 (1.13, 1.34) | **<0.001** | 1.09 (1.02, 1.18) | | **0.019** |
|  | **Race/Ethnicity** |  | *P for interaction = 0.695* | | *P for interaction = 0.316* | | |
|  | Mexican American | 755 | 1.18 (1.03, 1.35) | **0.016** | 1.12 (0.97, 1.29) | | 0.129 |
|  | Non-Hispanic Asian people | 760 | 1.18 (1.06, 1.31) | **0.003** | 1.24 (0.94, 1.62) | | 0.119 |
|  | Non-Hispanic Black people | 1130 | 1.14 (1.05, 1.23) | **0.003** | 1.05 (0.95, 1.16) | | 0.333 |
|  | Non-Hispanic White people | 1926 | 1.19 (1.11, 1.28) | **<0.001** | 1.07 (0.98, 1.16) | | 0.142 |
|  | Other Hispanic or Multi-Racial | 788 | 1.24 (1.12, 1.36) | **<0.001** | 1.24 (1.10, 1.39) | | **<0.001** |
| **VA** | **Overall** | **5359** | 5.08 (4.08, 6.33) | **<0.001** | 1.63 (0.93, 2.84) | | 0.085 |
|  | **Age (Quartiles)** |  | *P for interaction = 0.537* | | ***P for interaction = 0.048*** | | |
|  | Q1 (<30 years old) | 1412 | 4.31 (2.91, 6.38) | **<0.001** | 5.57 (1.36, 22.82) | | **0.018** |
|  | Q2 (30~40 years old) | 1339 | 5.95 (4.09, 8.66) | **<0.001** | 2.76 (1.36, 5.59) | | **0.006** |
|  | Q3 (40~50 years old) | 1369 | 7.03 (4.82, 10.25) | **<0.001** | 2.82 (1.40, 5.68) | | **0.005** |
|  | Q4 (>50 years old) | 1239 | 5.36 (3.50, 8.20) | **<0.001** | 1.04 (0.52, 2.10) | | 0.909 |
|  | **Sex** |  | ***P for interaction = 0.003*** | | *P for interaction = 0.317* | | |
|  | Male | 2719 | 7.89 (5.74, 10.86) | **<0.001** | 1.74 (0.73, 4.17) | | 0.208 |
|  | Female | 2640 | 3.86 (3.01, 4.97) | **<0.001** | 1.32 (0.65, 2.69) | | 0.431 |
|  | **Race/Ethnicity** |  | *P for interaction = 0.473* | | *P for interaction = 0.157* | | |
|  | Mexican American | 755 | 5.62 (2.97, 10.62) | **<0.001** | 1.69 (0.28, 10.35) | | 0.553 |
|  | Non-Hispanic Asian people | 760 | 8.04 (5.05, 12.81) | **<0.001** | 7.07 (1.69, 29.61) | | **0.009** |
|  | Non-Hispanic Black people | 1130 | 3.72 (2.45, 5.65) | **<0.001** | 2.50 (1.23, 5.05) | | **0.012** |
|  | Non-Hispanic White people | 1926 | 5.16 (3.99, 6.67) | **<0.001** | 1.28 (0.60, 2.75) | | 0.514 |
|  | Other Hispanic or Multi-Racial | 788 | 4.93 (2.83, 8.59) | **<0.001** | 2.06 (0.92, 4.63) | | 0.079 |

Note: All regression models were estimated using Firth logistic regression. All models were adjusted for age (except age-stratified models), sex (except sex-stratified models), education, smoking, drinking status, physical activity, education level, family history of cardiovascular disease and family history of stroke. Age quartiles are defined as follows: Q1 (Lowest) represents the youngest 25% of the population, Q2 the 25–50% range, Q3 the 50–75% range, and Q4 (Highest) the oldest 25%. Established CKM Status: Mid (Stage 2)/Late (Stage 3-4) vs. Early (Stage 0-1). Advanced CKM Status: Late (Stage 3-4) vs. Early (Stage 0-1)/Mid (Stage 2).
Abbreviations: CI, Confidence Interval; CKM, Cardiovascular-Kidney-Metabolic; IRD-PS, Insulin Resistance/Dyslipidemia Pathological Score; OR, Odds Ratio; SLI-PS, Systemic Low-Grade Inflammation Pathological Score; VA, Visceral Adiposity.

**Supplementary Table S14. Exploratory Decomposition of VA Associations Through IRD-PS and SLI-PS in Shaanxi Sample**

| **Subgroup** | **Decomposition Pathway** | **Established CKM Status** | | | **Advanced CKM Status** | | |
| --- | --- | --- | --- | --- | --- | --- | --- |
|  |  | **Estimate (95% CI)** | ***P* value** | **Prop. Attenuated** | **Estimate (95% CI)** | ***P* value** | **Prop. Attenuated** |
| **Sex** | | | | | | | |
| Male | VA → IRD-PS → Outcome | 0.0173  (0.0123, 0.0229) | **<0.001** | 62.85 | -0.0009  (-0.0031, 0.0012) | 0.404 | -8.77 |
|  | VA → SLI-PS → Outcome | 0.0007  (-0.0005, 0.0021) | 0.262 | 6.72 | 0.0008  (-0.0003, 0.0022) | 0.162 | 6.42 |
|  | Direct Estimate | 0.0102  (0.0001, 0.0215) | **0.048** |  | 0.0108  (0.0028, 0.0181) | **0.004** |  |
|  | Total Estimate | 0.0275  (0.0160, 0.0395) | **<0.001** |  | 0.0099 (0.0020, 0.0167) | **0.012** |  |
| Female | VA → IRD-PS → Outcome | 0.0128  (0.0098, 0.0160) | **<0.001** | 68.36 | 0.0018  (-0.0001, 0.0037) | 0.066 | 34.08 |
|  | VA → SLI-PS → Outcome | 0.0012  (-0.0002, 0.0027) | 0.104 | 15.52 | 0.0016  (0.0002, 0.0033) | **0.020** | 32.01 |
|  | Direct Estimate | 0.0057  (-0.0015, 0.0129) | 0.126 |  | -0.0001  (-0.0076, 0.0069) | 0.966 |  |
|  | Total Estimate | 0.0186  (0.0112, 0.0253) | **<0.001** |  | 0.0017  (-0.0053, 0.0082) | 0.576 |  |
| **Age (Quartiles)** | | | | | | | |
| Q1 (<31 years) | VA → IRD-PS → Outcome | 0.0111  (0.0072, 0.0156) | **<0.001** | 93.49 | -0.0009  (-0.0036, 0.0016) | 0.474 | 9.21 |
|  | VA → SLI-PS → Outcome | -0.0002  (-0.0027, 0.0022) | 0.832 | -1.78 | -0.0003  (-0.0031, 0.0023) | 0.802 | 3.85 |
|  | Direct Estimate | 0.0002  (-0.0097, 0.0097) | 0.910 |  | -0.0061  (-0.0191, 0.0044) | 0.286 |  |
|  | Total Estimate | 0.0113  (0.0011, 0.0199) | **0.026** |  | -0.0070  (-0.0202, 0.0038) | 0.240 |  |
| Q2 (31~42) | VA → IRD-PS → Outcome | 0.0168  (0.0116, 0.0228) | **<0.001** | 52.36 | 0.0004  (-0.0016, 0.0025) | 0.640 | 6.09 |
|  | VA → SLI-PS → Outcome | 0.0024  (0.0005, 0.0050) | **0.006** | 11.93 | -0.0004  (-0.0019, 0.0008) | 0.564 | -4.56 |
|  | Direct Estimate | 0.0152  (0.0052, 0.0245) | **0.002** |  | 0.0060  (-0.0002, 0.0096) | 0.054 |  |
|  | Total Estimate | 0.0320  (0.0226, 0.0395) | **<0.001** |  | 0.0064  (0.0009, 0.0093) | **0.036** |  |
| Q3 (42~55) | VA → IRD-PS → Outcome | 0.0195  (0.0130, 0.0264) | **<0.001** | 58.37 | 0.0034  (0.0004, 0.0068) | **0.036** | 21.09 |
|  | VA → SLI-PS → Outcome | -0.0002  (-0.0026, 0.0022) | 0.876 | -1.22 | 0.0012  (-0.0007, 0.0037) | 0.214 | 7.90 |
|  | Direct Estimate | 0.0142  (0.0003, 0.0293) | **0.048** |  | 0.0122  (0.0018, 0.0200) | **0.028** |  |
|  | Total Estimate | 0.0337  (0.0177, 0.0482) | **<0.001** |  | 0.0156  (0.0067, 0.0225) | **0.002** |  |
| Q4 (>55 years) | VA → IRD-PS → Outcome | 0.0046  (0.0001, 0.0107) | **0.042** | 23.00 | -0.0022  (-0.0059, 0.0013) | 0.222 | -12.11 |
|  | VA → SLI-PS → Outcome | 0.0019  (-0.0002, 0.0058) | 0.068 | 11.46 | 0.0040  (0.0011, 0.0085) | **0.002** | 15.74 |
|  | Direct Estimate | 0.0167  (-0.0020, 0.0492) | 0.104 |  | 0.0200  (0.0028, 0.0403) | **0.024** |  |
|  | Total Estimate | 0.0213  (0.0012, 0.0559) | **0.032** |  | 0.0178  (0.0018, 0.0380) | **0.030** |  |

Note: All regression models were estimated using Firth logistic regression unless otherwise stated. Models were adjusted for age (except age-stratified models), sex (except sex-stratified models), education, smoking, drinking status, physical activity, education level, family history of cardiovascular disease and family history of stroke.

Abbreviations: CI, Confidence Interval; CKM, Cardiovascular-Kidney-Metabolic; IRD-PS, Insulin Resistance/Dyslipidemia Pathological Score; SLI-PS, Systemic Low-Grade Inflammation Pathological Score; VA, Visceral Adiposity.

**Supplementary Table S15. Exploratory Decomposition of VA Associations Through IRD-PS and SLI-PS in the NHANES**

| **Subgroup** | **Decomposition Pathway** | **Established CKM Status** | | | **Advanced CKM Status** | | |
| --- | --- | --- | --- | --- | --- | --- | --- |
|  |  | **Estimate (95% CI)** | **P value** | **Prop. Attenuated** | **Estimate (95% CI)** | **P value** | **Prop. Attenuated** |
| **Sex** | | | | | | | |
| Male | VA → IRD-PS → Outcome | 0.0007  (0.0001, 0.0021) | **<0.001** | 65.28% | 0.0014  (-0.0041, 0.0150) | 0.440 | 14.21% |
|  | VA → SLI-PS → Outcome | 0.0014  (-0.0048, 0.0076) | 0.584 | 2.01% | 0.0007  (-0.0019, 0.0054) | 0.284 | 7.89% |
|  | Direct Estimate | 0.0003  (0.0001, 0.0009) | **<0.001** |  | -0.0018  (-0.0411, 0.0048) | 0.352 |  |
|  | Total Estimate | 0.0010  (0.0002, 0.0030) | **<0.001** |  | -0.0005  (-0.0349, 0.0047) | 0.228 |  |
| Female | VA → IRD-PS → Outcome | 0.0105  (0.0043, 0.0219) | **<0.001** | 63.32% | 0.0040  (0.0001, 0.0187) | **0.016** | 19.88% |
|  | VA → SLI-PS → Outcome | 0.0078  (0.0024, 0.0142) | **0.008** | 12.39% | 0.0024  (0.0000, 0.0099) | 0.036 | 9.95% |
|  | Direct Estimate | 0.0056  (0.0031, 0.0095) | **<0.001** |  | -0.0070  (-0.0608, 0.0035) | 0.840 |  |
|  | Total Estimate | 0.0161  (0.0075, 0.0312) | **<0.001** |  | -0.0030  (-0.0436, 0.0044) | 0.596 |  |
| **Age (Quartiles)** | | | | | | | |
| Q1 (≤30) | VA → IRD-PS → Outcome | 0.0054  (0.0009, 0.0150) | **<0.00**1 | 68.54% | 0.0001  (-0.0000, 0.0017) | 0.956 | 0.82% |
|  | VA → SLI-PS → Outcome | 0.0043  (-0.0012, 0.0122) | 0.100 | 9.12% | -0.0000  (-0.0005, 0.0002) | 0.644 | -3.25% |
|  | Direct Estimate | 0.0020  (0.0006, 0.0042) | **0.004** |  | 0.0000  (0.0000, 0.0008) | **0.048** |  |
|  | Total Estimate | 0.0074  (0.0017, 0.0184) | **<0.001** |  | 0.0001  (0.0000, 0.0016) | **0.008** |  |
| Q2 (30~40) | VA → IRD-PS → Outcome | 0.0023  (0.0004, 0.0082) | **<0.001** | 57.91% | -0.0004  (-0.0040, 0.0003) | 0.592 | -11.56% |
|  | VA → SLI-PS → Outcome | 0.0013  (-0.0038, 0.0067) | 0.532 | 2.51% | 0.0000  (-0.0000, 0.0003) | 0.256 | 5.81% |
|  | Direct Estimate | 0.0015  (0.0004, 0.0039) | **<0.001** |  | 0.0012  (0.0001, 0.0068) | **<0.001** |  |
|  | Total Estimate | 0.0038  (0.0008, 0.0128) | **<0.001** |  | 0.0008  (0.0001, 0.0026) | **<0.001** |  |
| Q3 (40~50) | VA → IRD-PS → Outcome | 0.0024  (0.0004, 0.0082) | **<0.001** | 65.31% | 0.0004  (-0.0000, 0.0025) | 0.132 | 13.42% |
|  | VA → SLI-PS → Outcome | 0.0061  (0.0003, 0.0147) | **0.048** | 8.15% | -0.0001  (-0.0017, 0.0007) | 0.700 | 2.30% |
|  | Direct Estimate | 0.0011  (0.0003, 0.0030) | **<0.001** |  | 0.0010  (0.0001, 0.0032) | **0.016** |  |
|  | Total Estimate | 0.0035  (0.0007, 0.0109) | **<0.001** |  | 0.0014  (0.0001, 0.0048) | **0.004** |  |
| Q4 (>50) | VA → IRD-PS → Outcome | 0.0082 (0.0008, 0.0280) | **<0.001** | 54.33% | 0.0079  (-0.0172, 0.0379) | 0.364 | 3.94% |
|  | VA → SLI-PS → Outcome | 0.0059  (-0.0016, 0.0173) | 0.120 | 6.06% | 0.0069  (0.0001, 0.0216) | **0.048** | -8.02% |
|  | Direct Estimate | 0.0060  (0.0008, 0.0166) | **<0.001** |  | -0.0342  (-0.1481, 0.0094) | 0.688 |  |
|  | Total Estimate | 0.0142  (0.0016, 0.0465) | **<0.001** |  | -0.0263  (-0.1368, 0.0095) | 0.912 |  |
| **Race/Ethnicity** | | | | | | | |
| Mexican American | VA → IRD-PS → Outcome | 0.0087  (0.0005, 0.0424) | **<0.001** | 71.82% | -0.0081  (-0.0633, 0.0031) | 0.300 | -13.21% |
|  | VA → SLI-PS → Outcome | 0.0053  (-0.0053, 0.0167) | 0.232 | 8.95% | 0.0016  (-0.0000, 0.0158) | 0.144 | 8.81% |
|  | Direct Estimate | 0.0018  (-0.0005, 0.0053) | 0.060 |  | -0.0089  (-0.1180, 0.0173) | 0.388 |  |
|  | Total Estimate | 0.0105  (0.0008, 0.0436) | **<0.001** |  | -0.0170  (-0.1592, 0.0032) | 0.608 |  |
| Non-Hispanic Asian people | VA → IRD-PS → Outcome | 0.0011  (0.0001, 0.0051) | **<0.001** | 68.39% | -0.0002  (-0.0017, 0.0007) | 0.700 | 7.15% |
|  | VA → SLI-PS → Outcome | 0.0023  (-0.0070, 0.0117) | 0.552 | 3.54% | 0.0002  (-0.0000, 0.0013) | 0.416 | 10.32% |
|  | Direct Estimate | 0.0004  (0.0000, 0.0013) | **0.012** |  | 0.0002  (0.0000, 0.0037) | **0.036** |  |
|  | Total Estimate | 0.0015  (0.0001, 0.0061) | **<0.001** |  | 0.0000  (0.0000, 0.0026) | **0.024** |  |
| Non-Hispanic Black people | VA → IRD-PS → Outcome | 0.0182  (0.0029, 0.0601) | **<0.001** | 50.47% | 0.0007  (-0.0000, 0.0045) | 0.072 | 13.04% |
|  | VA → SLI-PS → Outcome | 0.0069  (0.0009, 0.0178) | **0.028** | 7.96% | 0.0002  (-0.0005, 0.0016) | 0.576 | 2.32% |
|  | Direct Estimate | 0.0142  (0.0041, 0.0281) | **0.004** |  | 0.0016  (0.0001, 0.0051) | **0.040** |  |
|  | Total Estimate | 0.0324  (0.0073, 0.0770) | **<0.001** |  | 0.0023  (0.0001, 0.0070) | **0.016** |  |
| Non-Hispanic White people | VA → IRD-PS → Outcome | 0.0035  (0.0010, 0.0100) | **<0.001** | 62.71% | 0.0042  (-0.0033, 0.0245) | 0.320 | 13.58% |
|  | VA → SLI-PS → Outcome | 0.0041  (0.0001, 0.0084) | **0.048** | 5.96% | 0.0020  (-0.0005, 0.0092) | 0.164 | 4.55% |
|  | Direct Estimate | 0.0019  (0.0007, 0.0044) | **<0.001** |  | -0.0102  (-0.0885, 0.0044) | 0.852 |  |
|  | Total Estimate | 0.0054  (0.0016, 0.0142) | **<0.001** |  | -0.0060  (-0.0710, 0.0044) | 0.684 |  |
| Other/  Multi-Racial | VA → IRD-PS → Outcome | 0.0127  (0.0010, 0.0430) | **<0.001** | 78.43% | 0.0038  (0.0000, 0.0230) | **0.020** | 24.89% |
|  | VA → SLI-PS → Outcome | 0.0100  (-0.0050, 0.0302) | 0.176 | 14.43% | 0.0046  (0.0001, 0.0288) | **<0.001** | 33.88% |
|  | Direct Estimate | 0.0021  (-0.0031, 0.0064) | 0.108 |  | -0.0049  (-0.0776, 0.0041) | 0.524 |  |
|  | Total Estimate | 0.0147  (0.0016, 0.0441) | **<0.001** |  | -0.0011  (-0.0556, 0.0062) | 0.272 |  |

Note: All regression models were estimated using Firth logistic regression unless otherwise stated. Models were adjusted for age (except age-stratified models), sex (except sex-stratified models), race/ethnicity (except race-stratified models), smoking status, drinking status, physical activity, and education level. All estimates, 95% CIs, and P-values were generated using non-parametric bootstrapping (500 simulations). Established CKM Status: Mid (Stage 2)/Late (Stage 3-4) vs. Early (Stage 0-1). Advanced CKM Status: Late (Stage 3-4) vs. Early (Stage 0-1)/Mid (Stage 2).

Abbreviations: CI, Confidence Interval; CKM, Cardiovascular-Kidney-Metabolic; IRD-PS, Insulin Resistance/Dyslipidemia Pathological Score; SLI-PS, Systemic Low-Grade Inflammation Pathological Score; VA, Visceral Adiposity.

**Supplementary Table S16. Stage-Specific Associations Between Pathological Axes with CKM Stage**

| **Variables** | **Shaanxi Dataset** | | **NHANES** | |
| --- | --- | --- | --- | --- |
|  | **OR (95% CI)** | ***P* value** | **OR (95% CI)** | ***P* value** |
| **Established CKM Status** | | | | |
| VA  (harmonized) | 1.01 (1.00, 1.03) | 0.053 | 1.03 (1.02, 1.04) | **<0.001** |
| IRD-PS  (harmonized) | 2.54 (2.23, 2.92) | **<0.001** | 2.96 (2.64, 3.33) | **<0.001** |
| SLI -PS  (harmonized) | 1.09 (1.00, 1.19) | 0.051 | 1.05 (0.98, 1.13) | 0.159 |
| **Advanced CKM Status** | | | | |
| VA  (harmonized) | 1.01 (1.00, 1.03) | 0.056 | 1.01 (1.00, 1.03) | 0.057 |
| IRD-PS  (harmonized) | 1.03 (0.95, 1.11) | 0.483 | 1.10 (0.99, 1.21) | 0.065 |
| SLI -PS  (harmonized) | 1.11 (1.03, 1.19) | **0.008** | 1.10 (1.00, 1.20) | **0.039** |

Note: All regression models were estimated using Firth logistic regression. The ‘harmonized’ pathological axes were constructed using a standardized set of variables common to both datasets. Specifically: VA (harmonized) was based on Waist Circumference (z-scored). IRD-PS (harmonized) was derived from a Principal Component Analysis (PCA) combining the Triglyceride-glucose (TyG) index, the Triglyceride (TG) / High-Density Lipoprotein Cholesterol (HDL-C) ratio, and negated HDL-C. SLI-PS (harmonized) was derived from a PCA combining White Blood Cell count (WBC), Neutrophil count (NE), Platelet count (PLT), and Uric Acid (UA) concentration.

Models for the Shaanxi dataset were fully adjusted for age, sex, smoking status, drinking status, physical activity, education level, family history of cardiovascular disease, and family history of stroke. Models for the NHANES dataset were fully adjusted for age, sex, smoking status, drinking status, physical activity, education level, and race/ethnicity.

Established CKM Status: Mid (Stage 2)/Late (Stage 3-4) vs. Early (Stage 0-1). Advanced CKM Status: Late (Stage 3-4) vs. Early (Stage 0-1)/Mid (Stage 2).

Abbreviations: CI, Confidence Interval; CKM, Cardiovascular-Kidney-Metabolic; HDL-C, High-Density Lipoprotein Cholesterol; IRD-PS, Insulin Resistance/Dyslipidemia Pathological Score; NE, Neutrophil Count; OR, Odds Ratio; PLT, Platelet Count; SLI-PS, Systemic Low-grade Inflammation Pathological Score; TG, Triglycerides; TyG, Triglyceride-Glucose Index; UA, Uric Acid; VA, Visceral Adiposity; WBC, White Blood Cell Count.

# **Supplementary Figures**


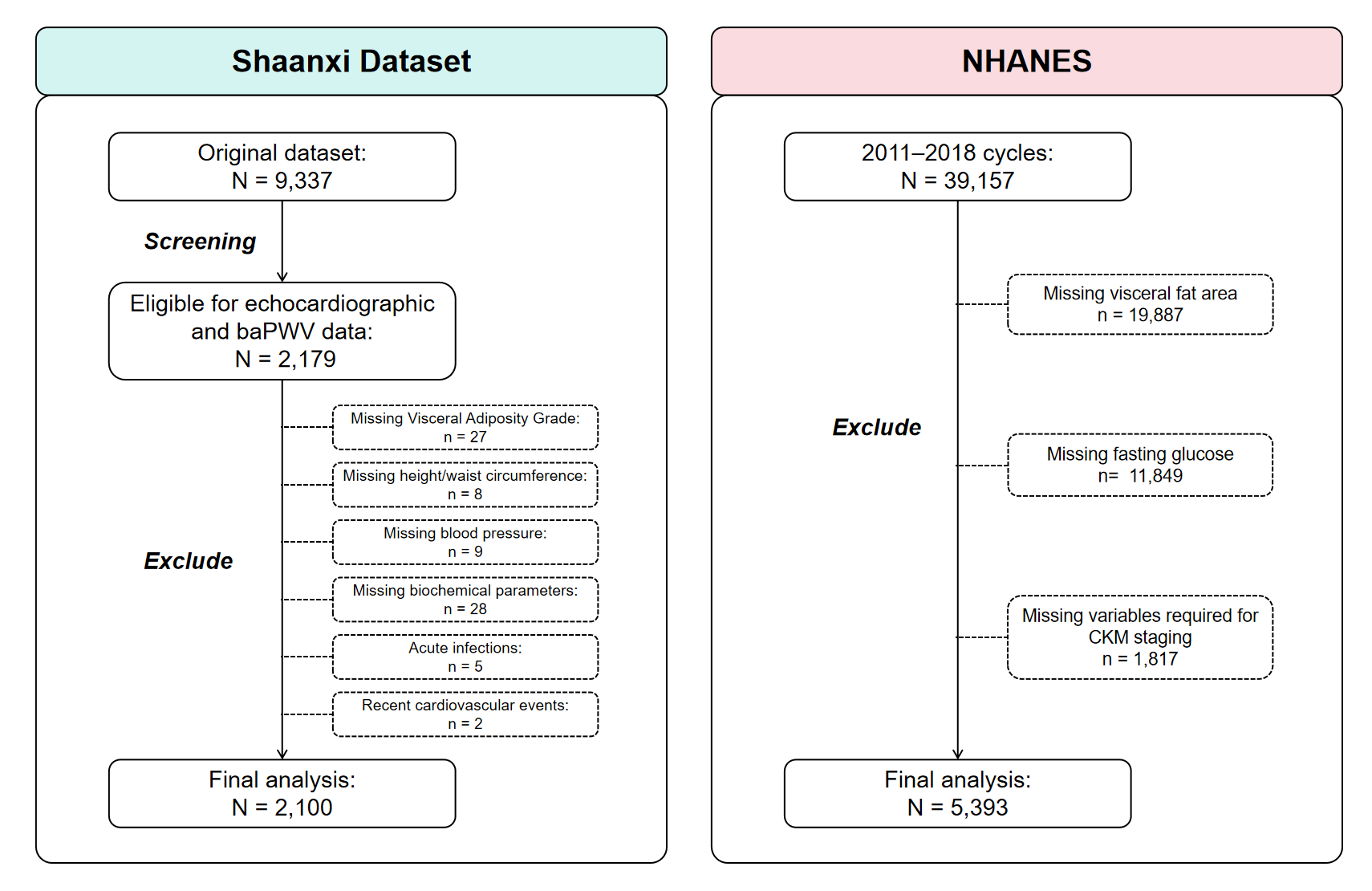


**Supplementary Figure S1. Study participant flow diagram illustrating inclusion and exclusion criteria for the Chinese Shaanxi dataset (N = 2,100) and NHANES (N = 5,393).**

**
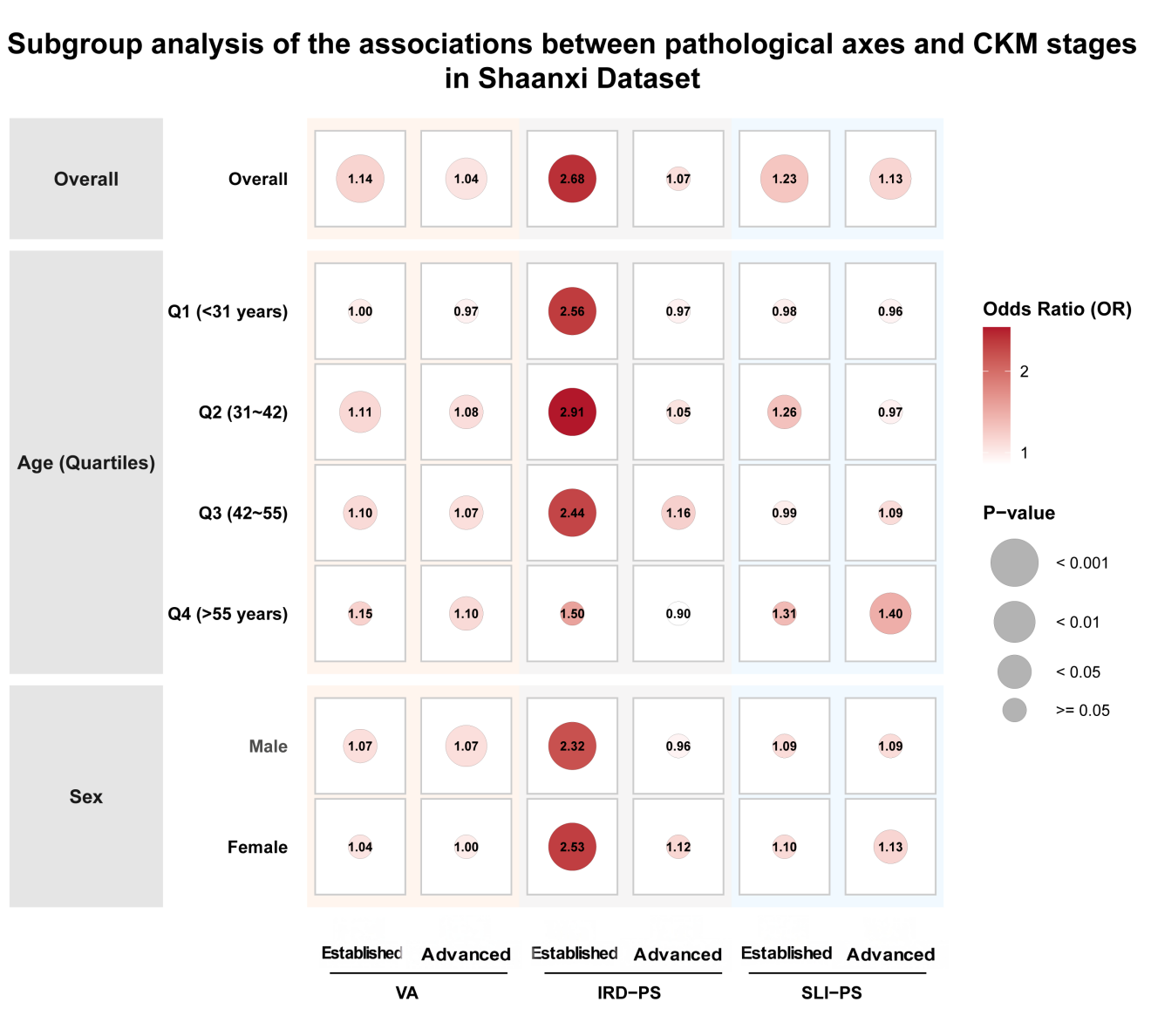
**

**Supplementary Figure S2. Subgroup analysis of the associations between pathological axes and CKM stages, stratified by age and sex in the Shaanxi dataset.**

Note: The figure displays the odds ratios (ORs) derived from fully adjusted Firth logistic regression models, corresponding to the data presented in Supplementary Table S10. The color intensity of each circle corresponds to the magnitude of the OR, with red indicating a positive association (OR > 1). The size of the circle corresponds to the statistical significance (*P* value).

Models were adjusted for age (except in age-stratified analyses), sex (except in sex-stratified analyses), smoking status, drinking status, physical activity, education level, family history of cardiovascular disease, and family history of stroke. Established CKM Status is defined as Mid (Stage 2)/Late (Stage 3-4) vs. Early (Stage 0-1). Advanced CKM Status is defined as Late (Stage 3-4) vs. Early (Stage 0-1)/Mid (Stage 2).

Abbreviations: CKM, Cardiovascular-Kidney-Metabolic; IRD-PS, Insulin Resistance/Dyslipidemia Pathological Score; OR, Odds Ratio; SLI-PS, Systemic Low-grade Inflammation Pathological Score; VA, Visceral Adiposity.

**
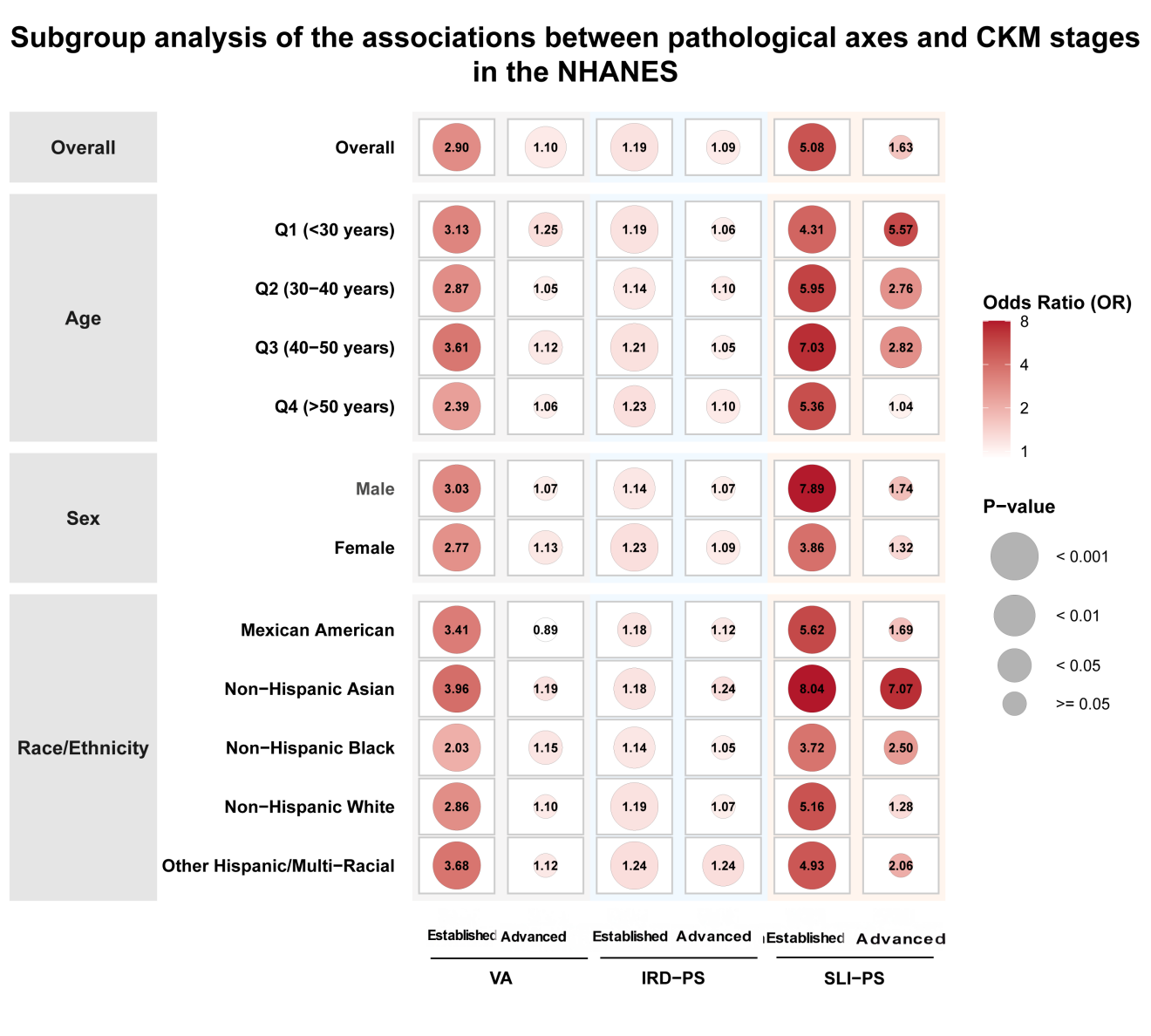
**

**Supplementary Figure S3. Subgroup analysis of the associations between pathological axes and CKM stages, stratified by age, sex, and race/ethnicity in the NHANES dataset.**

Note: The figure displays the odds ratios (ORs) derived from fully adjusted Firth logistic regression models, corresponding to the data presented in Supplementary Table S11. The color intensity of each circle corresponds to the magnitude of the OR, with red indicating a positive association (OR > 1). The size of the circle corresponds to the statistical significance (*P* value).

Models were adjusted for age (except in age-stratified analyses), sex (except in sex-stratified analyses), smoking status, drinking status, physical activity, and education level. Established CKM Status is defined as Mid (Stage 2)/Late (Stage 3-4) vs. Early (Stage 0-1). Advanced CKM Status is defined as Late (Stage 3-4) vs. Early (Stage 0-1)/Mid (Stage 2).

Abbreviations: CKM, Cardiovascular-Kidney-Metabolic; IRD-PS, Insulin Resistance/Dyslipidemia Pathological Score; OR, Odds Ratio; SLI-PS, Systemic Low-grade Inflammation Pathological Score; VA, Visceral Adiposity.


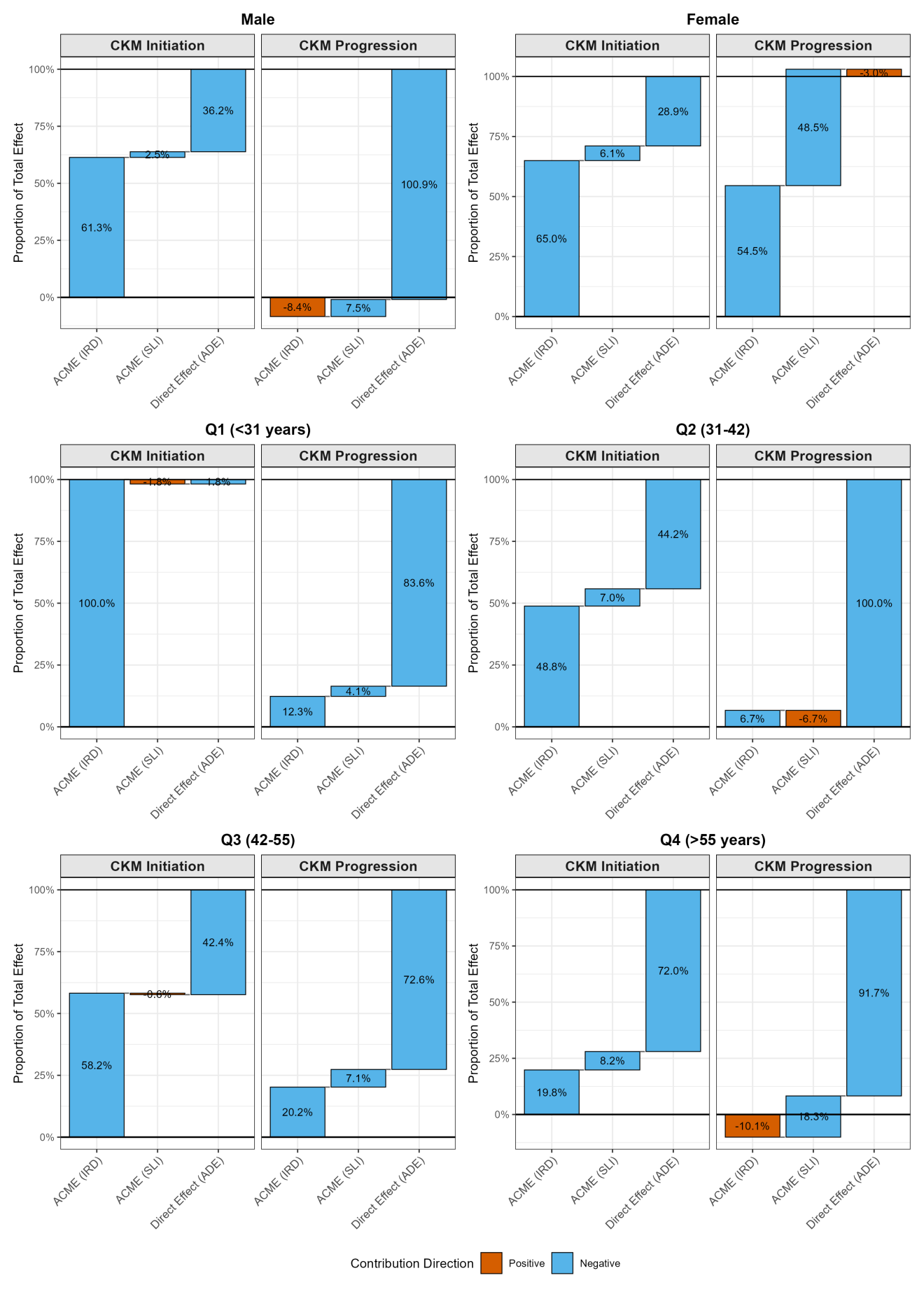


**Supplementary Figure S4. Waterfall plots of statistical attenuation proportions for the association of VA with CKM stages, stratified by sex and age in the Shaanxi dataset.**

Note: The waterfall plots illustrate the proportion of the total association of Visceral Adiposity (VA) with CKM stages that is statistically attenuated by IRD-PS (Attenuation Estimate (IRD)) or SLI-PS (Attenuation Estimate (SLI)), or attributable to the unattenuated direct association (Direct Estimate). Plots are stratified by CKM outcome (Established vs. Advanced CKM Status) and by subgroup (Sex and Age Quartiles) in the Shaanxi dataset. The direction of the contribution is indicated by color (blue = positive association, increasing risk; orange = inverse association, lower risk). All estimates are derived from fully adjusted models corresponding to the data in Supplementary Table S12.

Abbreviations: CKM, Cardiovascular-Kidney-Metabolic; IRD-PS, Insulin Resistance/Dyslipidemia Pathological Score; SLI-PS, Systemic Low-grade Inflammation Pathological Score; VA, Visceral Adiposity.


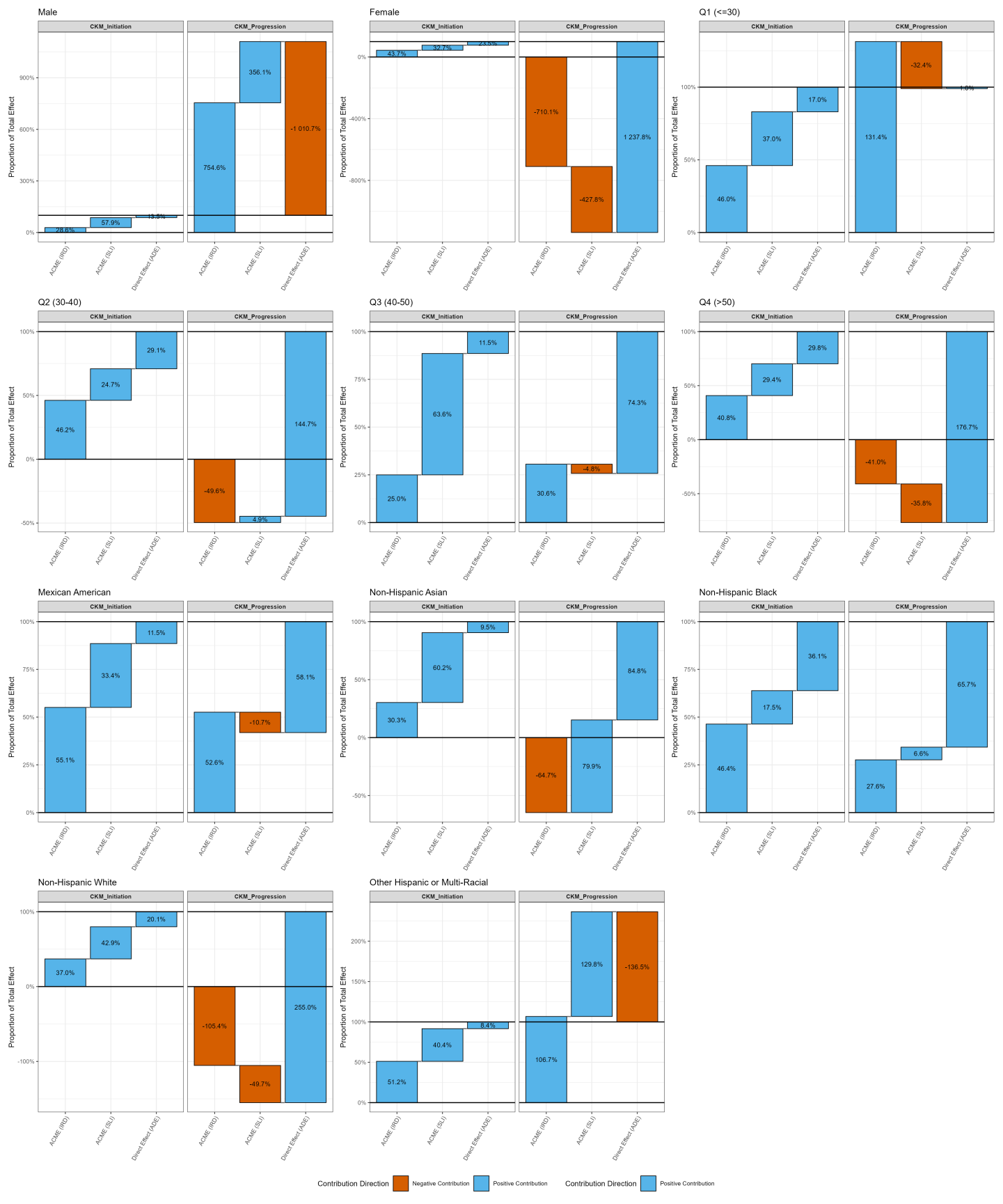


**Supplementary Figure S5. Waterfall plots of statistical attenuation proportions for the association of VA with CKM stages, stratified by sex, age, and race/ethnicity in the NHANES dataset.**

Note: The waterfall plots illustrate the proportion of the total association of Visceral Adiposity (VA) with CKM stages that is statistically attenuated by IRD-PS (Attenuation Estimate (IRD-PS)) or SLI-PS (Attenuation Estimate (SLI-PS)), or attributable to the unattenuated direct association (Direct Estimate). Plots are stratified by CKM outcome (Established vs. Advanced CKM Status) and by subgroup (Sex, Age Quartiles, and Race/Ethnicity) in the NHANES dataset. The direction of the contribution is indicated by color (blue = positive association, increasing risk; orange = inverse association, lower risk). All estimates are derived from fully adjusted models corresponding to the data in Supplementary Table S13.

Abbreviations: CKM, Cardiovascular-Kidney-Metabolic; IRD-PS, Insulin Resistance/Dyslipidemia Pathological Score; SLI-PS, Systemic Low-grade Inflammation Pathological Score; VA, Visceral Adiposity.
